# p21 restricts influenza A virus by perturbing the viral polymerase complex and upregulating type I interferon signaling

**DOI:** 10.1371/journal.ppat.1010295

**Published:** 2022-02-18

**Authors:** Chao Ma, Yuhan Li, Yanan Zong, Tony Velkov, Chenxi Wang, Xinyu Yang, Ming Zhang, Zhimin Jiang, Haoran Sun, Qi Tong, Honglei Sun, Juan Pu, Munir Iqbal, Jinhua Liu, Chongshan Dai, Yipeng Sun

**Affiliations:** 1 Key Laboratory of Avian Influenza of the Ministry of Agriculture and Key Laboratory of Animal Epidemiology of the Ministry of Agriculture, College of Veterinary Medicine, China Agricultural University, Beijing, China; 2 Department of Pharmacology & Therapeutics, School of Biomedical Sciences, Faculty of Medicine, Dentistry and Health Sciences, the University of Melbourne, Parkville, Victoria, Australia; 3 Department of Epidemiology and Biostatistics, University of Georgia, Athens, Georgia, United States of America; 4 The Pirbright Institute, Pirbright, Woking, United Kingdom; 5 Beijing Key Laboratory of Detection Technology for Animal-Derived Food Safety, College of Veterinary Medicine, China Agricultural University, Beijing, China; Icahn School of Medicine at Mount Sinai, UNITED STATES

## Abstract

Many cellular genes and networks induced in human lung epithelial cells infected with the influenza virus remain uncharacterized. Here, we find that p21 levels are elevated in response to influenza A virus (IAV) infection, which is independent of p53. Silencing, pharmacological inhibition or deletion of p21 promotes virus replication *in vitro* and *in vivo*, indicating that p21 is an influenza restriction factor. Mechanistically, p21 binds to the C-terminus of IAV polymerase subunit PA and competes with PB1 to limit IAV polymerase activity. Besides, p21 promotes IRF3 activation by blocking K48-linked ubiquitination degradation of HO-1 to enhance type I interferons expression. Furthermore, a synthetic p21 peptide (amino acids 36 to 43) significantly inhibits IAV replication *in vitro* and *in vivo*. Collectively, our findings reveal that p21 restricts IAV by perturbing the viral polymerase complex and activating the host innate immune response, which may aid the design of desperately needed new antiviral therapeutics.

## Author summary

Influenza A virus (IAV) poses a continuous threat to public health and economic stability. The ribonucleoprotein (RNP) of IAV is responsible for the transcription and replication of the viral RNA. These processes require interplay between host factors and RNP components. Here, we report that p21 can be activated by IAV infection and is controlled by a p53-independent pathway. We demonstrate that p21 directly binds to the viral polymerase acidic protein and limits IAV polymerase activity through disrupting the formation of the ribonucleoprotein complex. Additionally, p21 activation promotes IRF3 activation by blocking K48-linked polyubiquitination degradation of HO-1, thereby activating the type I interferon pathway. We further identify an 8-amino-acid peptide of p21 as the minimum motif that effectively inhibits IAV replication and presents therapeutic efficacy both *in vitro* and *in vivo*. Thus, our studies not only identify p21 as an antiviral protein, but also provide mechanistic insight to facilitate drug development.

## Introduction

Influenza A virus (IAV) is one of the most common respiratory tract pathogens, causing at least 500,000 deaths each year worldwide [1–4]. The influenza pandemic of 1918 killed more than 50 million people worldwide [5]. H1N1 and H3N2 are the dominant seasonal IAVs responsible for yearly epidemics that affect 5%–15% of the population, in which they cause upper respiratory tract infections [6]. H5NX and H7N9 avian influenza viruses are transmitted to humans and cause higher mortality rates of up to 40%–50% [7]. Over the past few decades, vaccines and antiviral drugs have achieved great success in the control and treatment of IAV infections; however, seasonal IAV still persists [8–10]. Therefore, new antiviral strategies, including different viral targets, cellular targets or immune-modulating drugs, are urgently needed.

The genome of IAV consists of eight single-stranded RNAs of negative polarity, present in the virions as ribonucleoproteins (RNPs); which encode at least 13 proteins [11–14]. Each viral RNA (vRNA) is encapsidated by multiple nucleoproteins (NPs) and is associated with a copy of the viral RNA-dependent polymerase (RdRp). The RdRp is a heterotrimeric complex of the polymerase basic 2 (PB2), polymerase basic 1 (PB1) and polymerase acidic (PA) proteins [15,16]. The efficiency of viral RNA synthesis, which is critical for virus propagation and pathogenicity, is dependent on RdRp activity [17]. Since the structure and activity of the viral RdRp is distinct from enzymes found in host cells, it is a promising drug target for the selective inhibition of viral replication.

In response to IAV infection, the host immune system is often activated to prevent viral replication [18]. Type I interferons (IFNs) are the main orchestrators of the antiviral innate immune response. This response establishes an antiviral state in both infected and neighboring cells by stimulating the expression of antiviral genes known as interferon-stimulated genes, including 2′,5′-oligoadenylate synthetase (OAS), myxovirus resistance protein 1 (Mx1) and interferon-stimulated gene 15 (ISG15) [19]. Heme oxygenase-1 (HO-1) is a critical early mediator of the innate immune response [20]. It is required for the activation of IFN regulatory factor 3 (IRF3) after viral infection. HO-1 specifically interacts with IRF3 and affects its phosphorylation and subsequent nuclear translocation, thereby reducing IFN production and the expression of primary IRF3 target genes, such as induced protein 10 (IP-10), regulated upon activation normal T cell expressed and secreted (RANTES) and monocyte chemoattractant protein 1 (MCP-1) [20].

Here, using RNAi library screening based on cellular transcription profiling, we found that the cyclin-dependent kinase inhibitor p21 (also known as p21^Waf1/Cip1^) was upregulated in human respiratory epithelial cells after IAV infection. p21 was demonstrated to act as a negative regulator of IAV replication. p21 plays critical roles in ensuring the expansion of cells in the innate immune system and maintaining balanced innate immune activities [21–26]. In animal models, p21 deficiency increases the lipopolysaccharide-induced activation of the nuclear factor kappa light chain enhancer of activated B cells (NF-κB) pathway and the production of proinflammatory cytokines during septic shock [27–29]. In addition, p21 appears to be a key regulator of HIV-1 infection in myeloid cells [30]. It has been reported that p21 can limit the replication of HIV-1 *in vitro* by blocking the biosynthesis of dNTPs and inhibiting the activity of CDK9, which are required in the process of viral reverse transcription and mRNA transcription [30–32]. Thus far, the role of p21 in IAV replication remains unclear.

In the present study, we showed that IAV infection induces p21 expression both in cell culture and in mice. We further demonstrated that p21 interacts with the *C*-terminus of IAV RdRp subunit PA to perturb the IAV polymerase complex, thereby limiting RdRp activity and subsequent viral replication. Additionally, PA-mediated upregulation of p21 promotes IRF3 activation by recruiting HO-1 through inhibiting K48-linked ubiquitination degradation, resulting in the increased expression of type I IFNs, which are the main orchestrators of the antiviral innate immune response. Furthermore, we showed that a synthetic peptide mimetic based on the 36 to 43 amino acid sequence of p21 protein significantly inhibited IAV replication.

## Results

### Cellular transcription profiling-based RNAi library screening for host factors regulating IAV infection

**Fig 1:**
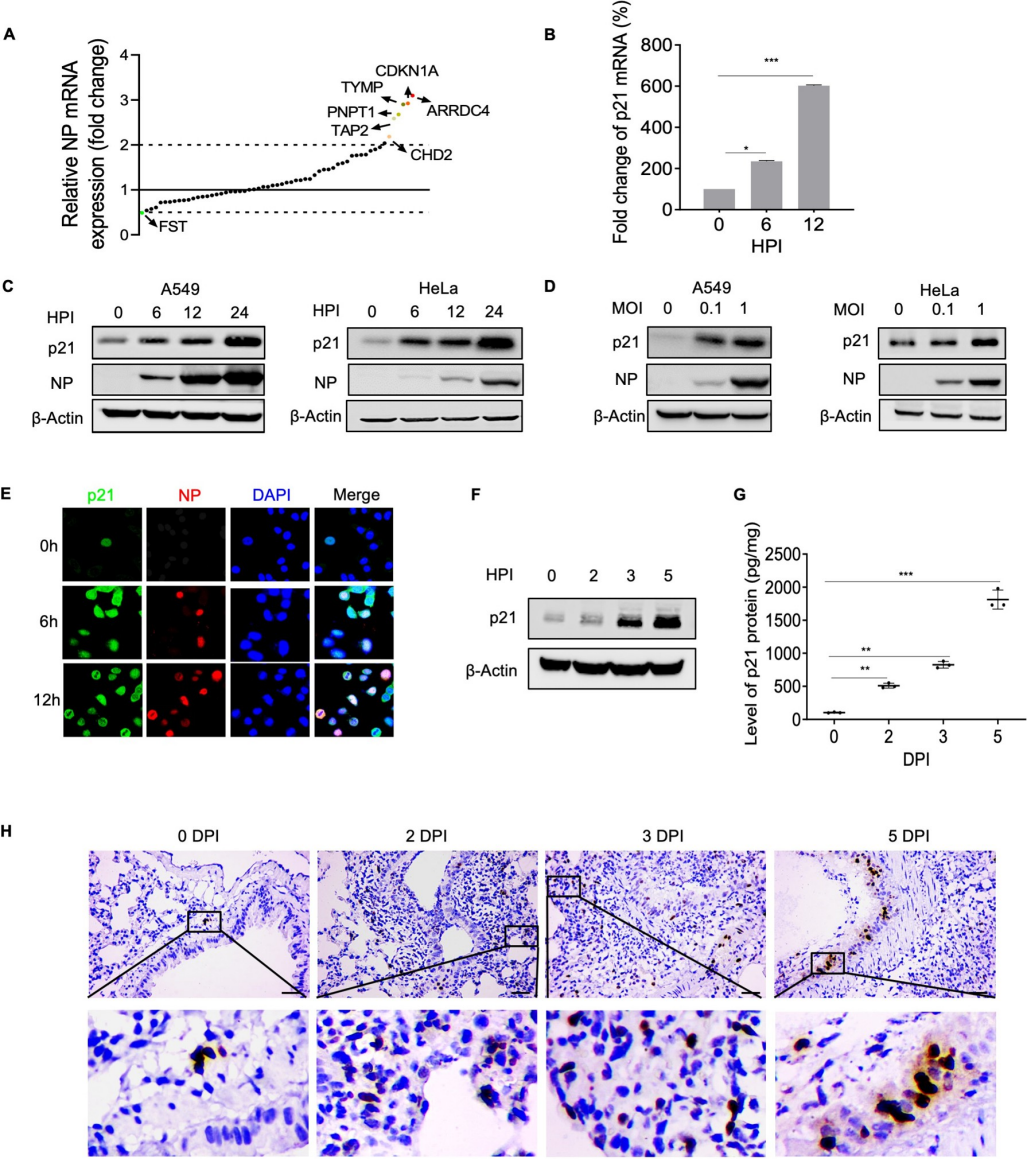
Identification of host factors that regulate IAV infection by cellular transcriptional profiling-based siRNA screening. (A) A549 cells were transfected with scrambled siRNA or the targeted siRNA library. The cells were infected with a 0.1 MOI of AH1 virus and cell lysates were analyzed by qRT-PCR. CDKN1A is also referred to as p21. (B) A549 cells were infected with AH1 viruses at a 0.1 MOI for different times. Total RNA was extracted and examined by RT-PCR. All data are expressed as the mean ± SEM and are representative of three independent experiments unless specified. (C) and (D) IAV infection upregulates the expression of p21 protein *in vitro*. A549 cells (C) or HeLa cells (D) were infected with AH1 virus and whole cell lysates were collected at the indicated time points to examine protein expression. (E) A549 cells were infected with AH1 virus, followed by staining with anti-p21 (green), NP (red) and DAPI nuclear stain (blue). Scale bar = 20 μm. (F to H) IAV upregulates p21 protein expression in mice. Mice were inoculated with AH1 viruses at 100 TCID_50_. p21 protein expression in the lung tissues was determined by western blotting (F), ELISA (G) and immunohistochemistry (H) at the indicated time points (n = 6 mice in each group). Scale bar = 100 μm. All data are representative or presented as the mean ± SEM of three independent experiments unless specified. *P < 0.05, **P < 0.05, ***P < 0.001.

We combined cellular transcriptional profiling with a small interfering RNA (siRNA) screen to identify host factors involved in IAV replication. Human lung epithelial cells (A549) were infected with H5N1 IAV A/Anhui/1/2005 (AH1) for 18 h, then cells were harvested, total RNA was isolated and RNA-Seq was performed. The results showed that 314 genes were significantly differentially expressed in IAV-infected A549 cells, of which 252 genes were upregulated and 62 genes were downregulated (**S1A, S1B and S9 Figs**). On the basis of a threshold fold change of Z > 2.0 and a *P*-value < 1 × 10^˗90^, we further conducted an siRNA screen in A549 cells using a library against the top 60 upregulated genes that had not been previously defined; viral RNA copies were quantified by qRT-PCR as the endpoint. Our screen revealed six host factors (ARRDC4, CDKN1A [p21^Waf1/Cip1^], TYMP, PNPT1, TAP2 and CHD2) as potential positive regulators and one host factor (FST) as a negative regulator of IAV replication (based on a > 2-fold change threshold). Notably, we observed that p21 knockdown significantly upregulated the expression of NP mRNA in AH1 virus-infected A549 cells (**Fig 1A).** To confirm the induction of p21 in response to IAV infection, A549 cells were infected with AH1 virus, then, the expression of p21 mRNA was determined at different time points post-infection. The results showed that the mRNA levels of p21 increased ~3–6-fold at 6 and 12 hours post-infection (hpi) (**Fig 1B**). Western blotting demonstrated that AH1 virus infection upregulated the expression of p21 protein at 6, 12 and 24 hpi in both A549 and HeLa cells, whereas IAV inactivated with ultraviolet light was unable to upregulate p21 (**Figs 1C and** S1**C**). In addition, p21 protein levels were elevated in a dose-dependent manner relative to the viral infection load (**Fig 1D**). Immunofluorescence assays also confirmed that p21 protein was significantly induced after AH1 infection (**Fig 1E**). To determine the expression profiles of p21 in response to influenza infection *in vivo*, C57BL/6J mice were infected intranasally with AH1 virus. Western blotting results showed that AH1 virus infection upregulated p21 protein levels in the lung tissue of mice at 2, 3 and 5 days post-infection (dpi) (**Fig 1F**). Similarly, ELISA and immunohistochemistry results demonstrated that AH1 virus infection upregulated the expression of p21 protein in the lung tissues of mice at 3 and 5 dpi (**Fig 1G and 1H**). Immunohistochemistry showed that the upregulation of p21 protein mainly occurred in bronchial and alveolar wall cells, which are the initial infective target cells of IAV. Taken together, these data showed that AH1 virus infection significantly upregulated the expression of p21 protein in multiple cell lines and in a murine animal model.

### p21 suppresses IAV replication *in vitro* and *in vivo*

**Fig 2:**
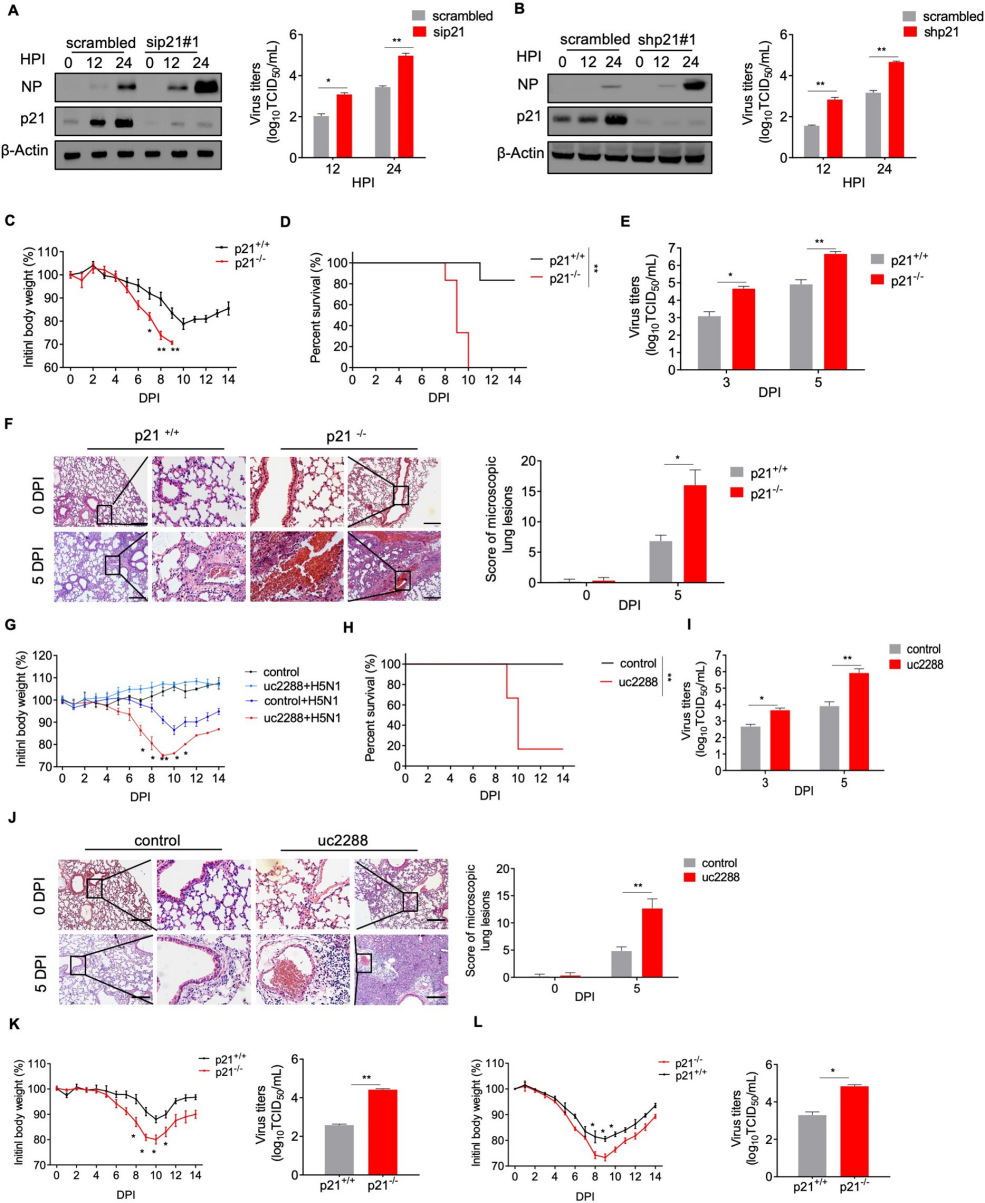
p21 is required for suppressing IAV replication *in vitro* and *in vivo*. (**A**) and (**B**) p21 knockdown increased IAV replication in A549 cells. (A) A549 cells treated with sicontrol or siRNA#1 were infected with AH1 virus at a 0.1 MOI. (B) p21-stable knockdown HeLa cells (shRNA#1) or control cells (scrambled) were infected with AH1 virus. Cell lysates were collected and analyzed by western blotting, and the virus yields of progeny were determined by a TCID_50_ assay. (**C** to **E**) p21-knockout (KO) mice were more susceptible to influenza virus pathology. Groups of wild-type (WT) or p21^˗/˗^ mice were infected with AH1 virus, and their body weights (C) and survival (D) were recorded for 14 days. Viral titers (E) were quantified in lung homogenates by a TCID_50_ assay at 3 and 5 dpi (n = 6 mice in each group). (**F**) H&E staining of the lungs of WT and p21^˗/˗^ mice. (**G** to **I**) C57BL/6J mice were orally gavaged with UC2288 or corn oil every other day. Then, mice were infected with influenza AH1 virus, and their body weights and survival were recorded for 14 days. Viral titers were quantified by a TCID_50_ assay at 3 and 5 dpi (n = 6 mice in each group). Scale bar = 100 μm. (**J**) H&E staining of the lungs of WT or UC2288-treated mice. (**K** and **L**) p21^+/+^ (i.e., WT) or p21^˗/˗^ mice were infected with H7N9 or H1N1 virus and their body weights were recorded for 14 days. Viral titers were quantified in lung homogenates as the TCID_50_ value at 5 dpi (n = 6 mice in each group). Scale bar = 100 μm. For **A** and **B**, data are representative or presented as the mean ± SEM of three independent experiments. *P < 0.05, **P < 0.05.

These initial observations led to our working hypothesis that p21 may affect influenza viral replication. To this end, we demonstrated that transfection of cells with p21-targeting siRNA (#1, #2, #3) and p21-targeting shRNA (#1, #2) significantly decreased p21 protein levels and promoted the expression of viral NP protein (**S2A and S2B Fig**). There was no significant effect on cell viability or cell growth rate with the knockdown of p21 post IAV infection (**S2C and S2D Fig**). Furthermore, treatment of the AH1 virus-infected A549 cells with p21 siRNA#1 significantly increased viral NP levels and virus titers at 12 and 24 hpi (**Fig 2A**). We also generated a p21-stable knockdown (shRNA#1) in HeLa cells and found that NP levels and the titers of AH1 virus were dramatically increased compared with native HeLa cells (**Fig 2B**). Similarly, silencing p21 protein also increased the expression of NP and the virus titer in NHBE cells after AH1 infection (**S2E Fig**). We then employed a pharmacological approach using a specific p21 protein inhibitor, UC2288, to confirm the inhibitory effect of p21 against IAV. UC2288 (5 μM) treatment significantly increased NP levels and the virus titer in A549 cells after AH1 virus infection (**S2F Fig**). To further confirm the inhibitory role of p21 against virus replication, Flag-p21 plasmid was transfected into A549 cells and then the cells were infected with AH1 virus. There was no significant effect on cell viability or cell growth rate with the overexpression of p21 upon IAV infection (**S2G Fig**). p21 overexpression resulted in almost complete viral inhibition, as noted by significantly decreased NP levels and virus titers at 24 hpi (**S2H Fig**). Similarly, HeLa cells transfected with Flag-p21 plasmid displayed decreased levels of viral protein production (**S2I Fig**). We also evaluated the role of p21 protein in the replication of other subtypes of IAV, including H7N9, H3N2 and H1N1. The results showed that transfection with p21-targeting siRNA significantly increased the NP level at 12 and 24 hpi (**S2J Fig**). To pin-point the effect of p21 on the transcription and replication of IAV, A549 cells with p21-knockdown were infected with AH1 virus, then vRNA and mRNA derived from NP were measured by qRT-PCR. At both time points, the levels of all viral RNAs were found to be significantly increased in the p21-knockdown cells compared with those in the control cells (**S2K Fig**). These findings indicate that p21 could inhibit the replication of IAVs of different subtypes (including H5N1, H7N9, H3N2 and H1N1) *in vitro*.

In the following studies, we evaluated the impact of p21 on the pathogenesis of IAV infection in wild-type (WT) and p21^-/-^ mice. Mice were intranasally infected with AH1 virus and the body weights and survival rates were recorded across the experimental time course. The p21^˗/˗^ mice suffered significant loss in body weight and an increased mortality rate compared with WT mice (**Fig 2C and 2D**). The viral titers in the lung tissues of p21^˗/˗^ mice were significantly higher than those of WT mice at 3 and 5 dpi (**Fig 2E**), and caused more severe lung tissue damage and inflammation (**Fig 2F**). WT mice were orally administered (15 mg/kg) the p21 inhibitor UC2288 and then infected with AH1 virus. The UC2288-treated mice displayed markedly decreased p21 protein expression levels in their lung tissues (**S3A Fig**), decreased body weight and increased viral titers compared with the vehicle-treated mice (**Fig 2G–2I**). Histopathological analysis showed that p21 pharmacology inhibition promoted lung tissue damage and inflammation (**Fig 2J**). Consistent with this, after AH1 infection, the levels of interleukin-6 (IL-6), tumor necrosis factor-alpha (TNF-α) and interleukin-1β (IL-1β) significantly increased in the lung tissues of p21^˗/˗^ mice compared with WT mice (**S3B Fig**). Immunohistochemical staining showed that NP levels were increased in p21^˗/˗^ and UC2288-treated mice compared with WT mice (**S3C Fig**). Similar findings were observed in mice infected with H7N9 or H1N1 virus (**Fig 2K and 2l**). Taken together, these results suggested that the host factor p21 can inhibit IAV replication both *in vitro* and *in vivo*.

### p21 was upregulated following interaction with the PA protein of IAV

**Fig 3:**
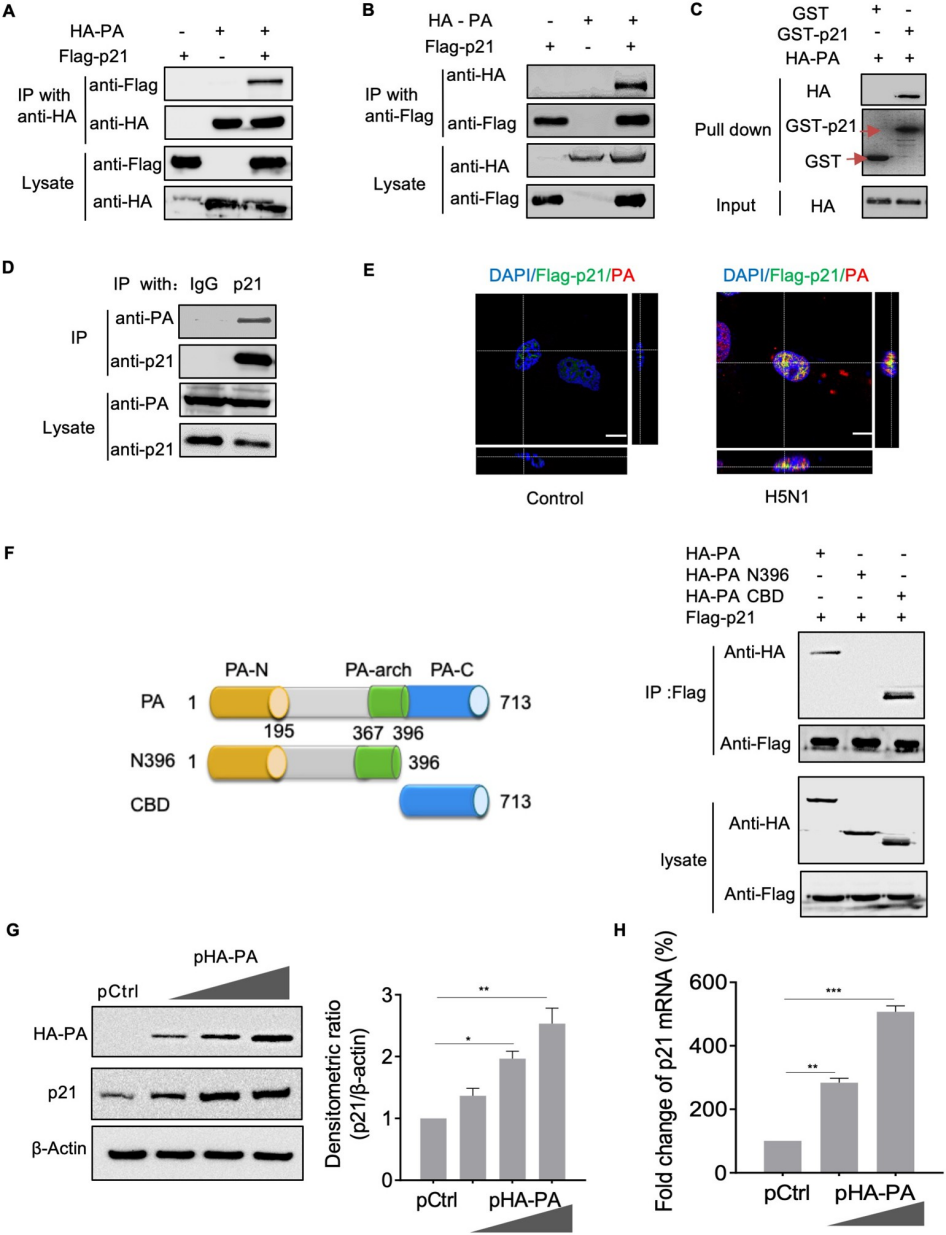
p21 was upregulated by interaction with the PA protein of IAV. (**A**) and (**B**) HEK293T cells were transfected individually or in combination with plasmids expressing Flag-p21 and HA-PA. Cell lysates were immunoprecipitated with an anti-HA (A) or anti-Flag mAb (B) and then analyzed by western blotting. (**C**) Lysates of *E*. *coli* transfected with the GST or GST-p21 construct were incubated with Glutathione Sepharose 4 Fast Flow and then mixed with lysates from cells transfected with HA-PA. Lysates were examined by western blotting and Coomassie blue staining. (**D**) Confluent A549 cells were infected with AH1 virus at a MOI of 1. Cell lysates were immunoprecipitated with anti-p21 polyclonal antibody and analyzed by western blotting. (**E**) A549 cells were transfected with Flag-p21 and then infected with AH1 virus, cells were determined by double-label indirect immunofluorescence with anti-Flag and anti-PA antibodies. Scale bar = 8 μm. (**F**) Flag-tagged p21 plasmids were individually transfected into HEK293T cells together with HA-PA-mutant plasmids. Cell lysates were subjected to immunoprecipitation and further analyzed by western blotting. (**G**) and (**H**) A549 cells were transfected with different amounts of PA expression vectors and the protein or mRNA expression of p21 was detected. For **A–G**, data are representative of three independent experiments. For **H**, data are presented as the mean ± SEM from three independent experiments. *P < 0.05, **P < 0.05, ***P < 0.001.

To study the mechanisms by which p21 inhibits IAV replication, firstly we identified the interactions between p21 and viral proteins that are effective for virus replication in infected cells. HEK293T cells were transfected with Flag-tagged p21 and HA-tagged viral proteins (i.e., PB2, PB1, PA, NP, M1 and NS1) individually or in combination. The results showed that p21 specifically interacts with influenza PA but not with other viral proteins (PB1, PB2, NP, M1 and NS1) in HEK293T cells (**Figs 3A and** S4**A**). In a reciprocal immunoprecipitation assay, Flag-p21 was present in the HA immune precipitates (**Fig 3B**), indicating the presence of both p21 and PA in the same complex in the cell lysate. We demonstrated that p21 protein could bind to PA *in vitro* using a pull-down assay with GST-tagged p21 and HA-tagged PA proteins (**Fig 3C**). The interaction between p21 and PA in the nucleus of A549 cells infected by AH1 virus was also confirmed through immunoprecipitation immunocolocalization imaging studies (**Fig 3D and 3E**). Together, these results indicated that PA is the major binding partner of p21 during IAV infection. We further determined the domain/motif that is responsible for the PA–p21 interaction by generating two truncated PA constructs, which were fused to the *C*-terminus of the HA tag, and then examined for their interaction with p21 in HEK293T cells. Co-immunoprecipitation showed that PA-CBD exhibited strong binding to p21, while the *N*-terminal mutants of PA interacted poorly with p21 (**Fig 3F**). These results indicated that the *C*-terminal region of PA is critical for its interaction with p21. Furthermore, HA-tagged PA, expressed in A549 cells, significantly upregulated p21 mRNA and protein levels in a dose-dependent manner (**Fig 3G and 3H**). Additionally, we demonstrated that IAV infection still significantly upregulated p21 protein expression in p53-deleted or IFNR-knockout cells (**S4B and S4C Fig**), indicating that IAV infection-induced p21 activation is independent of p53 activation or type I IFN induction. Overexpression of HA-tagged PB2 protein did not upregulate the level of p21 protein in A549 cells (**S4D Fig**). Taken together, these data demonstrated that IAV-induced upregulation of p21 protein expression is induced by PA protein.

### p21 inhibits IAV polymerase activity by competing with PB1 for PA binding

**Fig 4:**
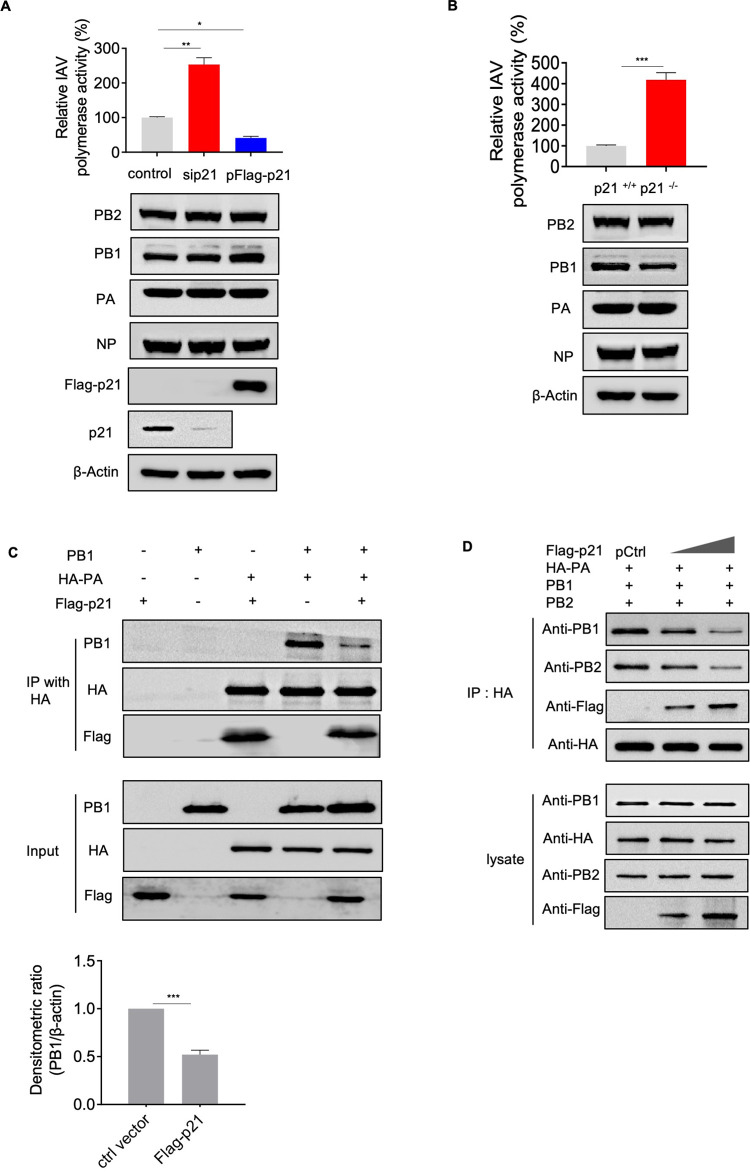
p21 inhibits IAV polymerase activity. (**A**) HEK293T cells treated with siRNA#1 or p21 expression plasmid were transfected with the four RNP protein expression constructs (PB2, PB1, PA and NP) derived from AH1 virus, together with pHH21-SC09NS F-Luc, which encodes NS vRNA possessing a reporter firefly luciferase gene. At 48 h post-transfection, a dual-luciferase assay was performed in which the relative firefly luciferase activity was normalized to the internal control, Renilla luciferase activity. (**B**) WT or p21-KO HEK293T cells were transfected with the four RNP protein expression constructs (PB2, PB1, PA and NP) derived from AH1 virus, together with pHH21-SC09NS F-Luc, which encodes NS vRNA possessing a reporter firefly luciferase gene. A dual-luciferase assay was performed. (**C** and **D**) p21 inhibits the interaction of PA and PB1. PB1, HA-PA and Flag-p21 were co-transfected into p21-KO HEK293T cells. After 48 h, cell lysates were harvested for immunoprecipitation. Data are representative or presented as the mean ± SEM of three independent experiments. *P < 0.05, **P < 0.05, ***P < 0.001.

The RdRp activity of IAV is one of the key factors that determines the efficiency of viral replication. IAV RdRp consists of three individual polypeptides (i.e., PB1, PB2 and PA), which are tightly intertwined [33]. During the process of influenza virus replication, the PA and PB1 subunits form a dimer in the cytoplasm before being efficiently imported into the nucleus by RanBP5 [34]. This dimer requires interactions between the *C*-terminus of PA and the *N*-terminus of PB1 [35]. In the present study, we found that p21 interacts with the *C*-terminal domain of PA (**Fig 3F**). Therefore, we hypothesized that p21 may compete with PB1 for PA binding to limit the RdRp activity of IAV. A luciferase reporter experiment was performed using the influenza virus minigenome assay to monitor viral polymerase activity. In brief, HEK293T cells were transfected with the indicated p21 or sip21 duplex, then cotransfected with PA, PB1, PB2, NP, pPolI-NS-firefly and pCMV-RL. At 48 h post-transfection, a dual-luciferase assay was performed in which the relative firefly luciferase activity was normalized to the internal control. The results showed that p21 knockdown significantly increased RNP activity to ~ 2.5-fold (**Fig 4A**), whereas p21 overexpression significantly decreased RNP activity to ~ 3-fold (**Fig 4A**). We constructed a p21-knockout (p21-KO) HEK293T cell line using the CRISPR/Cas9 system. The coding sequence of p21 was not detected, and the expression of p21 protein was not identified by western blotting in the knockout cell line (**S5A–S5D Fig**). At the same time, RdRp activity increased to ~ 4-fold in the p21-KO 293T cells (**Fig 4B**). Furthermore, we transfected the Flag-tagged p21, PA, PB1 and PB2 plasmids into p21-KO HEK293T cells. We found that p21 competed with PB1 for PA binding and reduced PB1 protein in the polymerase complex in a dose-dependent manner (**Fig 4C and 4D**), whereas the PB1–PB2 interaction remained intact (**S5E Fig**). This finding indicated that p21 reduces the incorporation of PB1 into the RdRp complex by competing for the PB1 binding motif at the *C*-terminus of PA, thereby disrupting the formation of the functional IAV polymerase complex.

### p21 positively regulates type I IFN expression upon IAV infection

**Fig 5:**
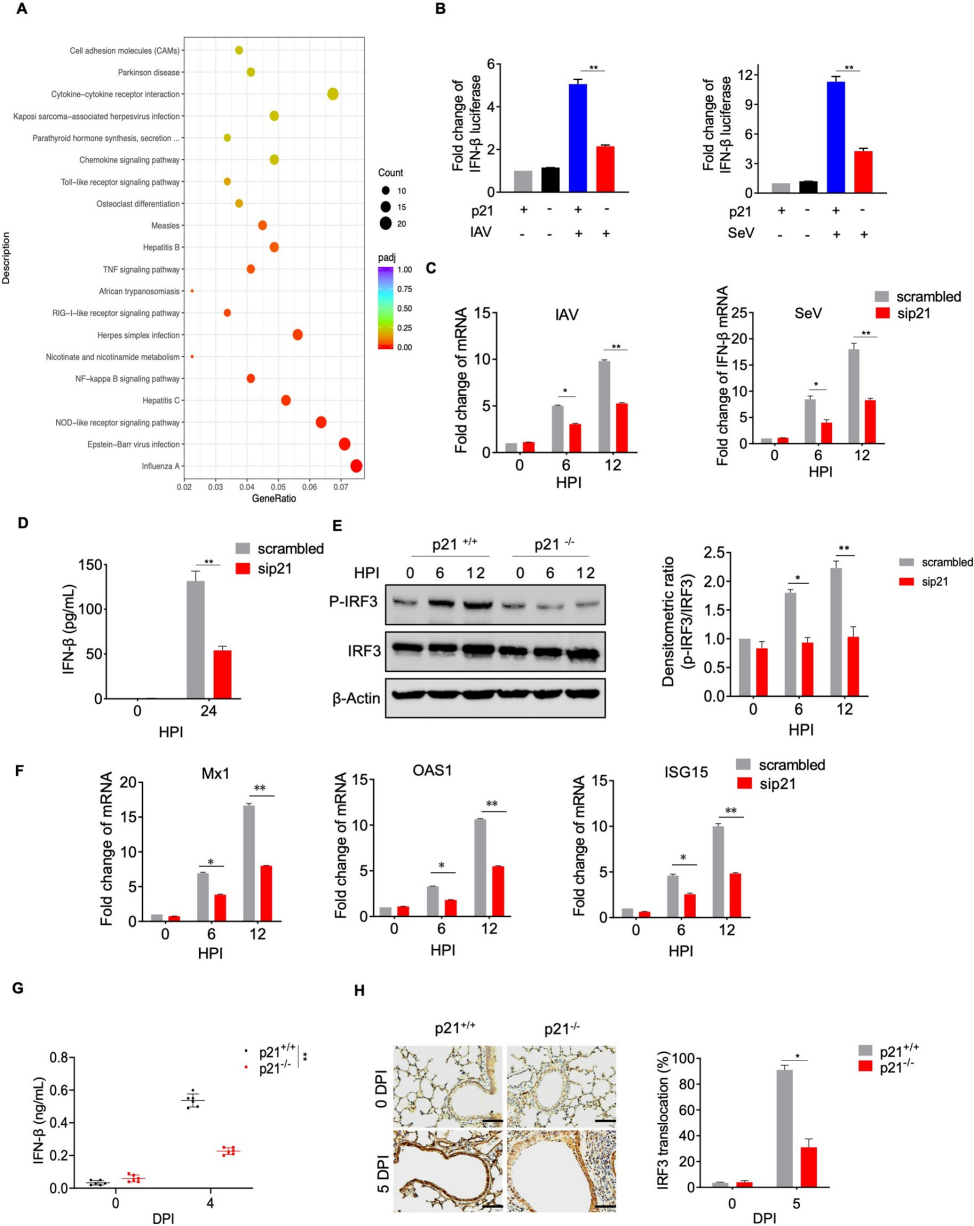
p21 positively regulates type I IFN expression upon IAV infection. (**A**) A549 cells treated with siControl or siRNA#1 were infected with AH1 virus at a 0.1 MOI. Cell lysates were collected and analyzed by transcriptome sequencing. (**B**) WT and p21-KO HEK293T cells were transfected with expression vectors for IFN firefly luciferase and TK-renilla luciferase. The transfected cells were treated with SeV or IAV for 16 h prior to the measurement of luciferase activity. (**C**) A549 cells treated with siControl or siRNA#1 were infected with AH1 or Sendai virus. Total RNA was harvested and analyzed by RT-qPCR. (**D**) A549 cells treated with siControl or siRNA#1 were infected with AH1 virus and the IFN-β levels were determined by ELISA. (**E**) WT and p21-KO HEK293T cells were infected with AH1 virus at a MOI of 1 and the levels of phosphorylated IRF3 were measured by western blotting. (**F**) A549 cells treated with siControl or siRNA#1 were infected with AH1 virus. Total RNA was harvested and analyzed by RT-qPCR. (**G** and **H**) WT and p21^˗/˗^ mice were challenged with AH1 virus at 100 TCID_50_, serum IFN-β levels were determined by ELISA (G), and the nuclear entry level of IRF3 in the lungs was analyzed by immunohistochemistry (H) (n = 6 mice in each group). All data are representative or presented as the mean ± SEM of three independent experiments unless specified. *P < 0.05, **P < 0.05, ***P < 0.001.

IAV infection can induce the expression of cytokines to initiate innate host defenses [36,37]. To identify the role of p21 protein in host defense during IAV infection, A549 cells were transfected with specific siRNA targeting p21 or with scrambled siRNA, and RNA-Seq was performed. KEGG pathway analysis (cut off *P*-value < 0.01 and |log_2_(fold change)| ≥ 1) found that silencing of p21 significantly changed various pathways, with the influenza A pathway being the most affected (**Fig 5A**). We further clustered the differentially expressed genes in the influenza A pathway and found that type I IFN-related genes were significantly reduced after p21 silencing during IAV infection (**S6 Fig**). Thus, we next compared IFN-β luciferase activity in WT and p21-KO HEK293T cells. The results showed that IFN-β luciferase activity induced by IAV and Sendai virus was remarkably inhibited in p21-KO HEK293T cells (**Fig 5B**). The qRT-PCR results showed that the abundance of IFN-β mRNA significantly decreased at 6 and 12 hpi in the p21-KO cells (**Fig 5C**). Consistent with this, the expression levels of IFN-β were significantly reduced in the p21-KO cells after AH1 virus infection (**Fig 5D**). IRF3 plays a critical role in the IFN-β response to viral infection [38]. We therefore evaluated p-IRF3 levels in WT and p21-KO HEK293T cells after AH1 virus infection. The results showed that the AH1 virus-induced increase in p-IRF3 expression was completely inhibited in p21-KO HEK293T cells compared with p21-WT HEK293T cells (**Fig 5E**). In addition, ISG-15, OAS1 and Mx1 expression was significantly decreased in p21-KO A549 cells after AH1 virus infection (**Fig 5F**). Consistent with this, the nuclear localization of IRF3 and serum IFN-β levels were significantly reduced in AH1-infected mice compared with p21-WT mice (**Fig 5G and 5H**). These results indicated that p21 deletion inhibits IRF3 nuclear activation and subsequently inhibits the production of IFN in mice. Taken together, our results suggested that p21 inhibition of IAV viral replication is partly dependent on the activation of IRF3 and type I IFN production.

### p21 promotes the activation of IRF3 by interacting with HO-1

**Fig 6:**
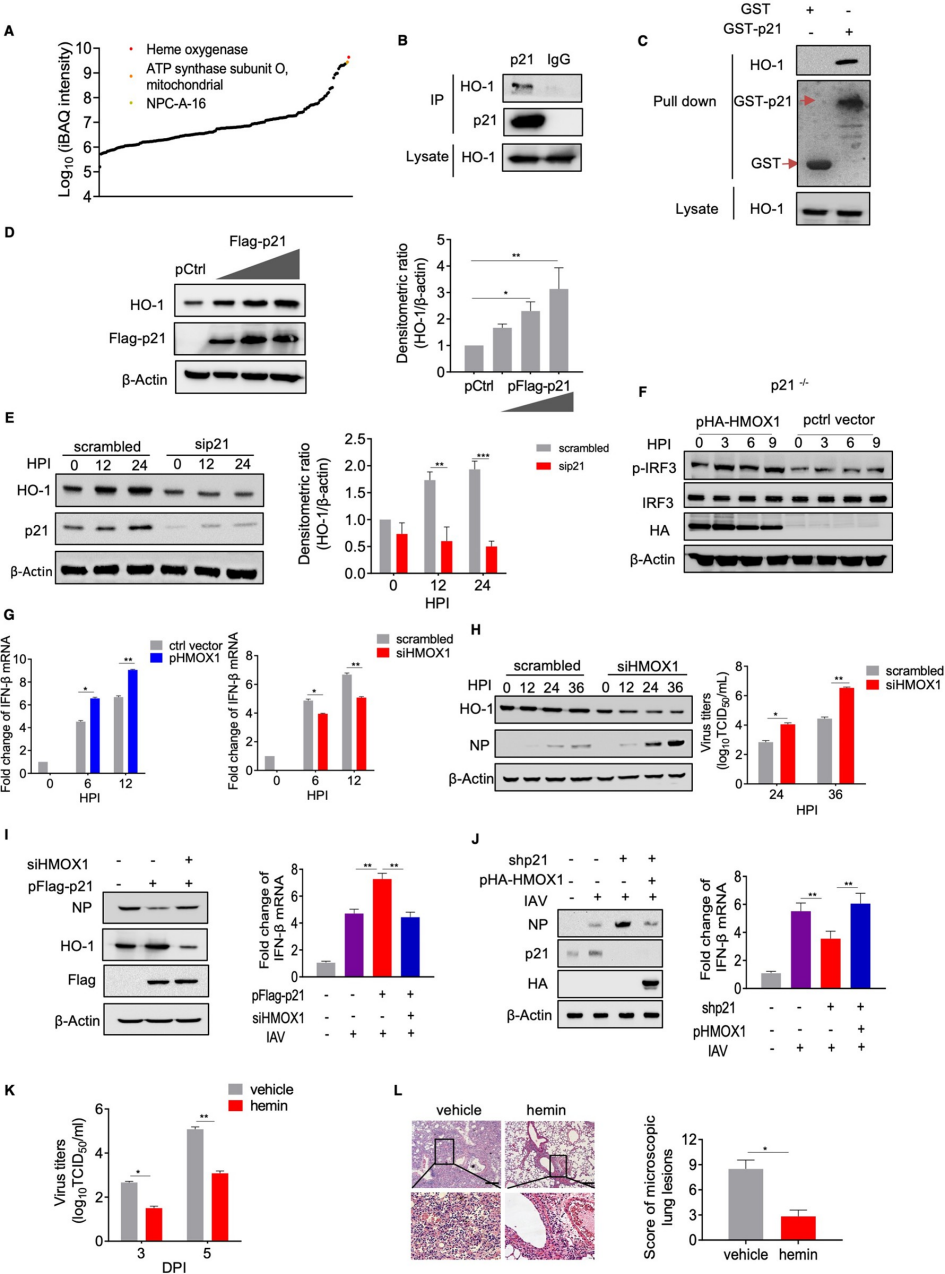
p21 interacts with HO-1 and inhibits its ubiquitination-mediated degradation. (A) A549 cells were infected with AH1 virus and immunoprecipitated with anti-p21 antibody or IgG. Protein analysis and identification were performed by mass spectrometry. (B) A549 cells were infected with AH1 virus at a MOI of 5. Cell lysates were immunoprecipitated and analyzed by western blotting. (C) Lysates of *E*. *coli* transfected with the GST or GST-p21 construct were incubated with Glutathione Sepharose 4 Fast Flow and then mixed with the p21 cell lysates. Lysates were examined by western blotting and Coomassie blue staining. (D) p21-KO HEK293T cells were transfected with different amounts of p21 expression vectors and the protein levels were detected by western blotting. (E) A549 cells treated with siControl or siRNA#1 were infected with AH1 virus at a MOI of 0.1. Cell lysates were collected and analyzed by western blotting. (F) p21-KO HEK293T cells were treated with p21 plasma or HMOX1 siRNA and infected with AH1 virus. Cell lysates were analyzed by western blotting or qPCR. (G) and (H) A549 cells were treated with HO-1 plasma or siRNA oligonucleotides and infected with AH1 virus. Cell lysates were collected and determined using qRT-PCR (G) or western blotting (H). Virus yields of the progeny were determined by the TCID_50_ assay. (I) A549 cells were treated with HO-1 plasma or the control and infected with AH1 virus. Cell lysates were analyzed by western blotting or RT-qPCR. (J) shRNA#1-HeLa cells were transfected with HO-1 plasma or the control and infected with AH1 virus. Cell lysates were analyzed by western blotting or RT-qPCR. (K and L) p21^˗/˗^ mice were orally gavaged with hemin or corn oil every other day and challenged with AH1 virus (n = 6 mice in each group). Viral titers and lung damage were quantified according to the TCID_50_ value and H&E staining. Scale bar = 100 μm. All data are representative or presented as the mean ± SEM of three independent experiments unless specified. *P < 0.05, **P < 0.05, ***P < 0.001.

To identify how p21 regulates the phosphorylation of IRF3, p21 protein complexes were first detected post IAV infection by affinity purification coupled with mass spectrometry analysis. Among all of the interacting proteins identified, we found that HO-1 was ranked in the top three in the screening list (**Fig 6A and S2 Table**). We then identified the interaction of p21 and HO-1 in A549 cells by co-immunoprecipitation. (**Fig 6B**). This interaction was further confirmed in a GST pull-down assay (**Fig 6C**). To investigate the functional significance of this interaction, we transfected the p21 expression plasmid into p21-KO HEK293T cells and detected the protein levels of HO-1. The data showed that the expression of p21 enhanced the levels of HO-1 protein in a dose-dependent manner (**Fig 6D**). Furthermore, p21 knockdown reduced the HO-1 protein levels in A549 cells after AH1 virus infection (**Fig 6E**). To examine whether p21 influences the stability of HO-1 protein after IAV infection, the half-life of p21 was evaluated using cycloheximide (CHX) and immunoblot analysis. We found that HO-1 had a shorter half-life in p21-KO HEK293T cells (**S7A Fig**). Similarly, overexpression of p21 significantly increased the half-life of HO-1 protein (**S7B Fig**). Furthermore, we showed that the ubiquitination of endogenous HO-1 with K48-linked chains, but not K63-linked chains, was significantly increased in p21-KO HEK293T cells post AH1 infection (**S7C Fig**). Collectively, our data shows that p21 binding to HO-1 reduces its K48-linked ubiquitination and inhibits its degradation post AH1 virus infection.

It has been previously reported that HO-1 is required for the activation of IRF3 after viral infection [20,39]. To determine whether HO-1 is involved in the regulation of IRF3 activation by p21 after IAV infection, we transfected the HO-1 expression plasmid into p21-KO HEK293T cells and assessed the activation level of IRF3 at different time points after virus infection. The results showed that p21 deletion inhibited the induction of IRF3 phosphorylation by AH1 virus, but this inhibitory effect was rescued in the presence HO-1 protein (**Figs 5E and 6F**). Consistent with this observation, we also found that overexpression of HO-1 significantly increased the levels of IFN-β and antiviral genes (including OAS1 and ISG15), whereas silencing of HO-1 decreased the levels of IFN-β and antiviral genes after virus infection (**Figs 6G and** S7**D**). Moreover, silencing of HO-1 significantly increased the levels of NP and the virus titer after AH1 virus infection (**Fig 6H**). To determine whether the regulation of IFN-β by p21 protein depends on HO-1 post IAV infection, we knocked down HO-1 in A549 cells overexpressing p21 protein and found that the expression of IFN-β was significantly reduced and the level of NP protein was increased (**Fig 6I**). Similarly, we transfected the HO-1 expression plasmid into stable p21-KO HeLa cells and found that overexpression of HO-1 significantly increased the levels of IFN-β and reduced NP protein expression (**Fig 6J**). To identify whether HO-1 activation can rescue the increased viral replication caused by p21 deficiency, p21^˗/˗^ mice were orally treated with the HO-1 inducer hemin (5 mg/kg), and the pulmonary virus titer was determined. Hemin treatment upregulated the expression of HO-1 protein and significantly reduced the expression of NP protein and the virus titer in the lung tissue compared with the vehicle-treated p21^˗/˗^ mice (**Figs 6K, S**7**E and S**7**F**). These results were confirmed by histopathological examination (**Fig 6l**). Collectively, these results provide solid evidence that p21^˗/˗^ mice are more susceptible to IAV infection, whereas HO-1 activation can rescue the increased viral replication caused by p21 deficiency.

### p21 peptide mimics inhibit IAV replication

**Fig 7:**
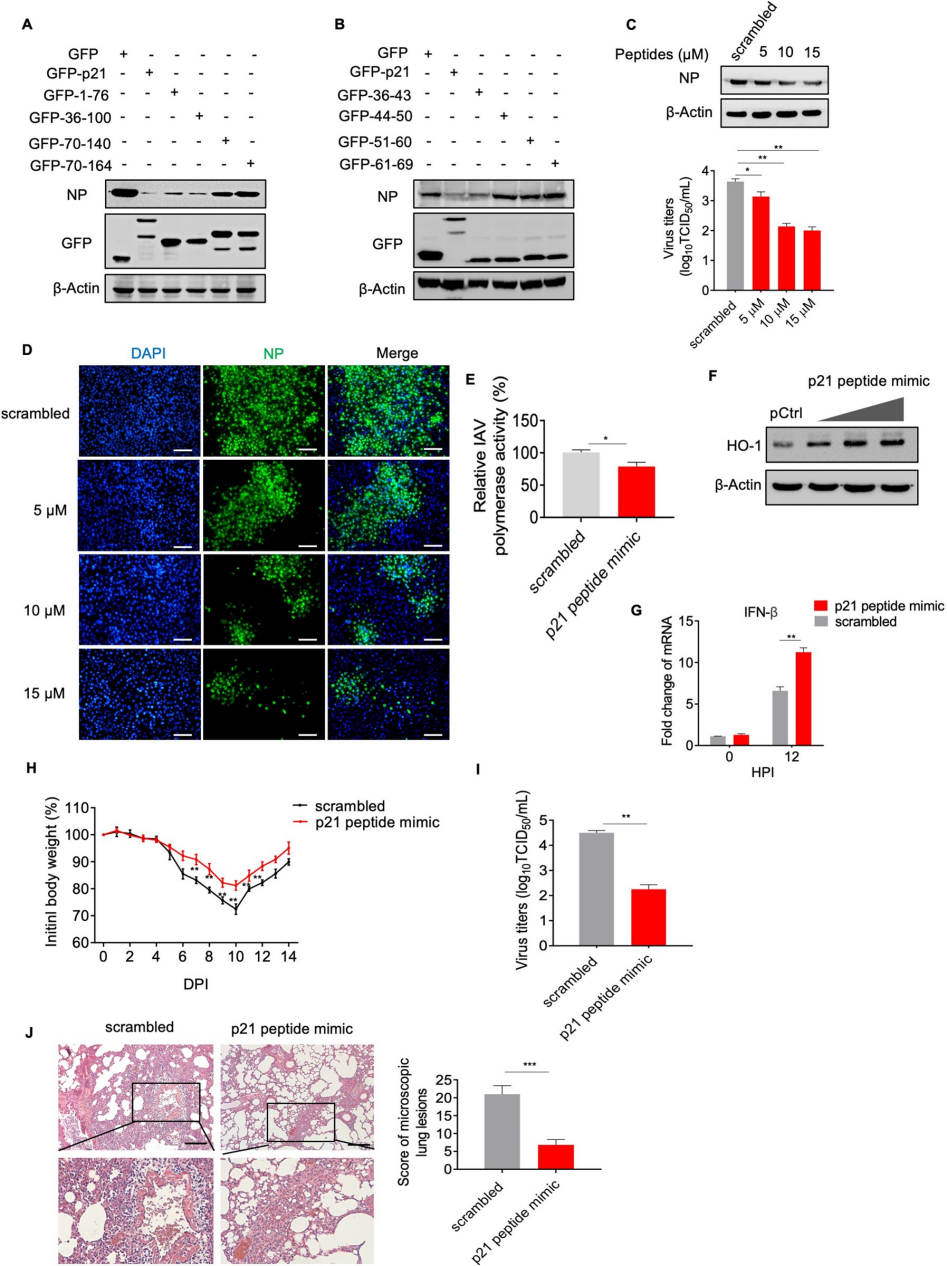
p21 peptide mimics inhibit IAV replication. (**A**) and (**B**) Identification of the amino acid sequence that inhibits IAV replication. A549 cells were transfected with GFP-p21 mutant plasmid and infected with a 0.1 MOI of AH1 virus. Cell lysates were collected and analyzed by western blotting. (**C**) p21 peptides mapping to residues x–y of the p21 protein inhibit IAV replication in cells. A549 cells were infected with a 0.1 MOI of AH1 virus and were treated with different concentrations of peptide mimics. Then, cell lysates were collected and analyzed by western blotting. (**D**) A549 cells were treated with different concentrations of peptide mimics and infected with AH1 virus, followed by staining with anti-NP (green) and DAPI nuclear stain (blue). Scale bar = 10 μm. (**E**) HEK293T cells were treated with the scramble or p21 peptide mimic and then transfected with the four RNP protein expression constructs and pHH21-SC09NS F-Luc. A dual-luciferase assay was performed in which the relative firefly luciferase activity was normalized to the internal control. (**F** and **G**) A549 cells were treated with different concentrations of peptide mimics and infected with AH1 virus. Cell lysates were collected and analyzed by western blotting or qRT-PCR. (**H** to **J**) C57/BL6J mice received an intraperitoneal injection of the scrambled peptides or p21 peptide mimics every other day and were then challenged with AH1 virus at 50 TCID_50_ (n = 6 mice in each group). Body weights (H) were recorded for 14 days. Viral titers (I) and lung damage (J) were quantified by the TCID_50_ value and H&E staining. Scale bar = 100 μm. All data are representative or presented as the mean ± SEM of three independent experiments unless specified. *P < 0.05, **P < 0.05, ***P < 0.001.

To identify the functional region of p21 protein that is crucial for the anti-IAV effects, we generated a series of truncated p21 constructs that were fused to the *N*-terminus of the GFP-tag, and then inoculated them into A549 cells infected with AH1 virus. The results showed that the amino acid sequence from position 36 to 43 of p21 protein conferred anti-IAV activity (**Fig 7A and 7B**). To further confirm the function of this amino acid sequence, p21 peptide mimics (36–43) were synthesized and their effects on the replication of IAV were tested in A549 cells. The results showed that the peptides inhibited NP expression in a dose-dependent manner, and the virus titers were significantly reduced in the peptide mimic-treated A549 cells (**Fig 7C**). Immunofluorescence assays also confirmed that NP protein expression was significantly inhibited in A549 cells treated with peptide mimics at 15 μM (**Fig 7D**). It is worth noting that, the peptide mimics did not affect the cell cycle or cell viability, indicating that there were no such side effects (**S8A and S8B Fig**). To determine the mechanism by which peptide mimics inhibit IAV replication, whether by affecting the activity of the viral polymerase or promoting the response of IFN-β to IAV infection, we tested the effect of peptide mimics on IAV polymerase and IFN pathways and found that they could inhibit the activity of the IAV polymerase (**Fig 7E**). Treatment with p21 peptide mimics also significantly increased the expression of HO-1 protein, then promoted the transcriptional activity of IFN-β after virus infection (**Fig 7F and 7G**).

To further evaluate whether the p21 peptide mimics could protect against IAV *in vivo*, mice were administered peptide mimics (15 mg/kg) intraperitoneally every 48 h. The scramble-treated mice suffered significant weight loss compared with the peptide mimic-treated mice (**Fig 7H**), and the viral titers in the lung tissues of scramble-treated mice were significantly higher than those in the peptide mimic-treated mice at 3 and 5 dpi (**Fig 7I**). Histopathological analysis showed that scramble-treated mice possessed more severe lung tissue damage and inflammation compared with peptide mimic-treated mice (**Fig 7J**). Furthermore, the peptide mimic-treated mice displayed markedly decreased NP protein expression levels in their lung tissue (**S8C Fig**). Collectively, these results showed that the amino acid sequence from position 36 to 43 of p21 plays an important role in inhibiting IAV replication, and the related peptide mimics exert therapeutic effects during influenza infection.

## Discussion

The interplay between IAV infection and the host is a dynamic process, which determines the pathogenicity and host range of the virus [40]. Some human genes associated with IAV infection have been identified on the basis of genome-wide RNAi library screens [41–46]. Zhou *et al*. found that FMRP is associated with A/Puerto Rico/8/34 (H1N1) virus NP/RNP nuclear export [45]. Karlas *et al*. showed that SON is important for the stable trafficking of A/WSN/33 (H1N1) virions to late endosomes early in infection and that CLK1 reduces IAV replication by impairing the splicing of viral M2 messenger RNA [46]. In the present study, we performed a siRNA screen using the H5N1 subtype. Our key findings revealed seven novel IAV-inducible genes that have not been reported previously. Among them, the *ARRDC4*, *CDKN1A*, *TYMP*, *PNPT1*, *TAP2* and *CHD2* genes restricted IAV infection, whereas *FST* promoted IAV infection. Further research on these genes may provide insight into new host antiviral mechanisms, or into how viruses hijack host proteins to escape the immune system. p21, encoded by the *CDKN1A* gene, is a member of the cyclin-dependent kinase inhibitor (CKI) family. It has been shown to regulate various cellular processes, including the cell cycle, DNA replication and repair, cell differentiation, apoptosis, and innate and adaptive immunity [26,47]. For the first time, we identified p21 as a negative regulator of IAV replication by combining cellular transcriptional profiling with siRNA screening. We found that p21 not only inhibited the replication of IAV in a variety of cell lines, but importantly, the titer of IAV increased significantly in p21^˗/˗^ mice and in animals orally treated with a specific p21 inhibitor. In addition, the antiviral protective effect of p21 was effective against different IAV strains and various subtypes including H1N1, H3N2, H5N1 and H7N9. Further analyses revealed that the 36 to 43 amino acid motif of p21 plays a critical role in limiting the replication of IAV. We synthesized peptides containing these amino acid sequences and found that the peptides possessed a significant inhibitory effect on IAV replication. In summary, our novel findings substantiate the inhibitory role of p21 against IAV infection and provide a theoretical basis for the development of anti-influenza drugs.

The replication and transcription of the IAV genome is catalyzed by the viral RNA polymerase [15]. This enzyme participates in the entire life cycle of the virus and is highly conservative in structure, having no host analogues, making it an attractive drug target. Fu *et al*. showed that TRIM32 could inhibit IAV polymerase activity by targeting PB1 for proteasome degradation, and thereby provides protection against IAV infection [48]. TRIM35 has also been found to defend the host against IAV infection by catalyzing K63-linked polyubiquitination of TRAF3 and activating RIG-I antiviral signaling [49]. Wang *et al*. demonstrated that plakophilin 2 binds to the *C*-terminal domain of PB1, thus blocking the interaction of PB2 with PB1 [50]. Our findings eluded to a novel host-directed mechanism whereby p21 directly binds to the PA subunit of the viral polymerase, disrupting the formation of IAV RdRp polymerase complexes and thereby inhibiting IAV replication. Specifically, we determined that p21 inhibits viral polymerase activity by interacting with the *C*-terminus of the PA subunit, thereby competing with the PB1 subunit for binding to this motif.

Humans have evolved a broad spectrum of innate immune defenses to limit IAV infection. These innate antiviral defenses indirectly inhibit infection by triggering signaling cascades that lead to the production of IFNs and other antiviral effector molecules [51]. We analyzed the differential gene expression in normal and p21-KO cells after influenza virus infection, and found that type I IFN signaling genes were significantly reduced after p21 knockdown. We identified a new role of p21 in innate immunity, whereby it acts as a positive regulator of type I IFN during IAV infection. HO-1 promotes the production of type I IFN and the expression of multiple IFN-stimulated genes by promoting IRF3 phosphorylation and increasing IRF3 signaling activation. Our results reveal that following IAV infection, p21 directly interacts with HO-1 to inhibit K-48 ubiquitination-mediated degradation.

**Fig 8:**
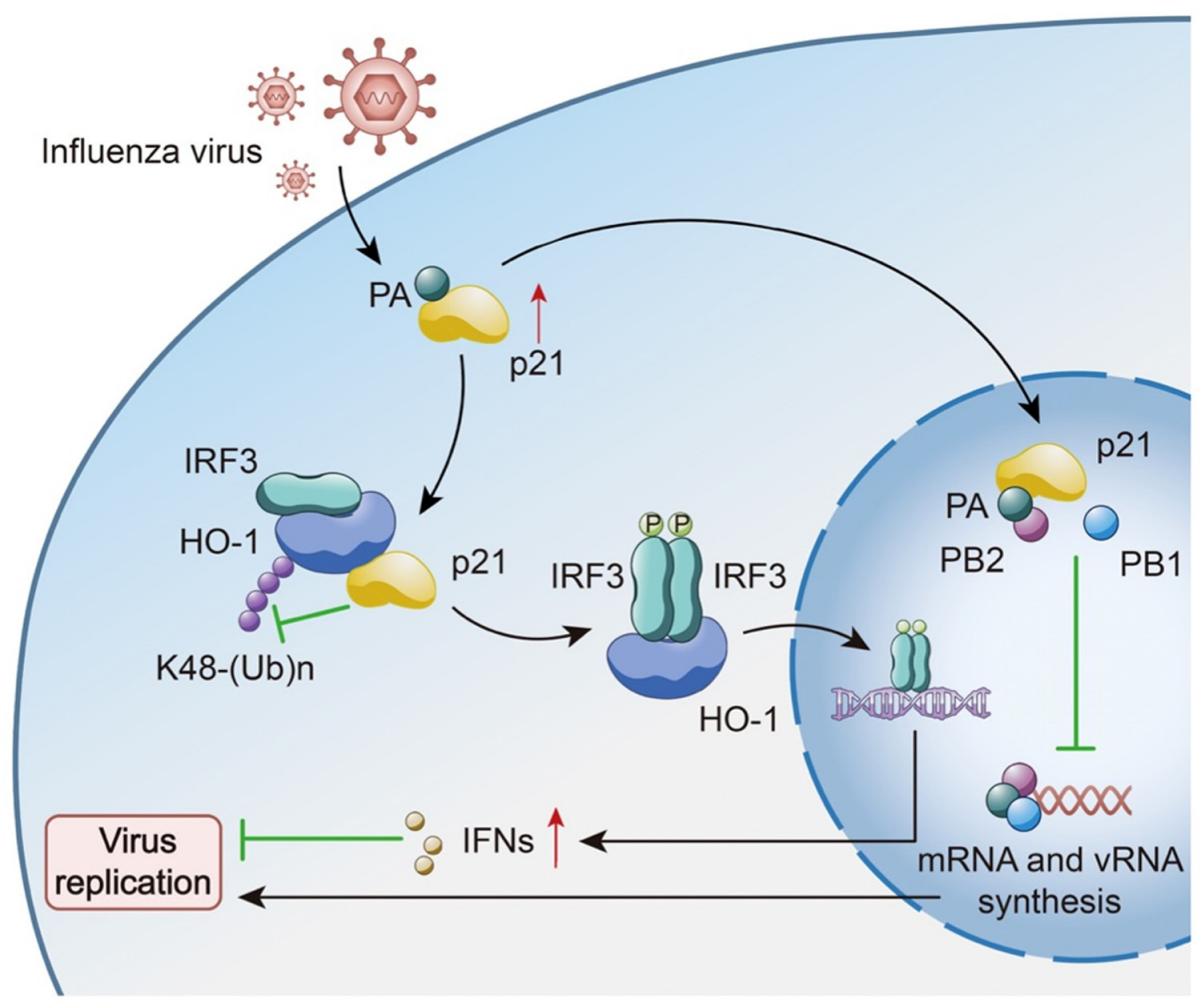
A proposed model. During IAV infection, p21 directly interacts with the C-terminus of the IAV polymerase subunit PA and competes for PB1 binding, which perturbs the formation of a functional polymerase complex and thereby inhibits viral replication. The interaction between p21 and PA proteins also enhances p21 expression, and then promotes IRF3 activation via the recruitment of HO-1, finally activating the expression of type I IFNs and exerting antiviral activity. p21-recruited HO-1 expression occurs through the inhibition of K48-linked ubiquitination degradation.

In the current study, our results suggested that p21 is an intrinsic IAV restriction factor, which exerts antiviral function through several complementary mechanisms. For the first time, our findings revealed that p21 interacts with the *C*-terminus of the IAV polymerase subunit PA and competes for PB1 binding, which perturbs the formation of a functional polymerase complex and thereby inhibits viral replication (**Fig 8**). Additionally, the interaction between the p21 and PA proteins enhances p21 expression. Moreover, we demonstrated that the antiviral activity of p21 is also mediated via its ability to promote IRF3 activation via the recruitment of HO-1 through the inhibition of K48-linked ubiquitination degradation, resulting in increased expression of type I IFNs (**Fig 8**). A synthetic peptide based on the 36 to 43 amino acid sequence of p21 markedly inhibited viral replication both *in vitro* and *in vivo*. Overall, our novel findings highlight the physiological role and crosstalk of p21 in antiviral innate immunity and provide a theoretical basis for future drug design.

## Materials and methods

### Ethics statement

All animal studies were performed in accordance with institutional guidelines of China Agricultural University (CAU) (approval SKLAB-B-2018-021) and approved by the Beijing Association for Science and Technology of China (approval SYXK, Beijing, 2007–0023).

### Viruses and cells

Influenza viruses A/California/04/2009 (H1N1), A/Anhui/1/2005 (AH1, H5N1), A/CK/BJ/FH620-L1/17 (H7N9) and A/Jiangxi/262/05 (H3N2) were maintained in our laboratory. Viruses were propagated in 10-day-old specific-pathogen-free embryonated eggs (Merial, Beijing, China). HEK293T cells (human embryonic kidney cell line), A549 cells (human lung adenocarcinoma epithelial cell line) and HeLa cells were obtained from the National Infrastructure of Cell Line Resource (Shanghai, China). All cells were maintained in Dulbecco’s modified Eagle’s medium (Life Technologies, Rockville, MD, USA) supplemented with 10% fetal bovine serum (Life Technologies), 100 U/mL penicillin and 100 μg/mL streptomycin at 37°C under a humidified atmosphere containing 5% CO_2_. NHBE cells (Lonza, Allendale, NJ, USA) were cultured in bronchial epithelial cell growth medium (Lonza) at the air–liquid interface, as previously described [49]. p53^˗/˗^ HCT116 cells were kindly provided by Dr Jun Tang (CAU, Beijing, China). All experiments with live viruses were performed in a biosafety level 3 laboratory.

### Antibodies

Primary antibodies were purchased from the following commercial suppliers: anti-p21 (#2947), anti-p-IRF3 (#37829), anti-K63-linkage specific polyubiquitin (#5621), anti-K48-linkage specific polyubiquitin (#8081) and anti-ubiquitin (#3936) antibodies were from Cell Signaling Technology (Beverly, MA, USA); anti-p21 (ab188224), Anti-p21 antibody [EPR3993] (ab109199) and anti-IRF3 (ab68481) were purchased from Abcam (Cambridge, UK); anti-HA (26183) and anti-Flag (MA1-91878) antibodies were purchased from Sigma-Aldrich (St Louis, MO, USA); anti-HO-1 (10701-1-AP) antibody was purchased from Proteintech (Wuhan, China); anti-NP monoclonal antibody (mAb) was purchased from GenScript (Nanjing, China); and anti-PA (GTX118991), anti-PB1 (GTX125923) and anti-PB2 (GTX125926) antibodies were purchased from GeneTex (Irvine, CA, USA). The secondary antibodies used in western blotting were goat anti-mouse IgG (072-07-18-06) and goat anti-rabbit IgG (072-07-15-06) purchased from KPL (Gaithersburg, MD, USA). The secondary antibodies used in confocal microscopy were Alexa Fluor 488 donkey anti-rabbit IgG (A21206) and Alexa Fluor 594 goat anti-mouse IgG (A11005) obtained from Life Technologies.

### Viral titration and growth kinetics in cells

The TCID_50_ was determined in MDCK cells by inoculation with 10-fold serially diluted viruses and incubation at 37°C for 72 h. The TCID_50_ value was calculated by the Reed–Muench method [50]. Multistep replication kinetics were determined using A549 and HeLa cells. A549 and HeLa cells were infected with virus, overlaid with serum-free DMEM containing 1 μg/mL tosylsulfonyl phenylalanyl chloromethyl ketone (TPCK)-trypsin (Sigma-Aldrich), and incubated at 37°C. NHBE cells were infected with virus and cultured in B-ALI growth medium (Lonza) at 37°C. The supernatants were sampled at 12 and 24 hpi and titrated by inoculating MDCK cells into a 96-well plate. Three independent experiments were performed.

### RNA interference

siRNA or shRNA targeting p21 or scrambled RNA at a concentration of 100 nM was transfected into A549 cells seeded in six-well plates using the Lipofectamine RNAiMAX transfection reagent (Waltham, MA, USA).

Lentivirus expressing p21-specific short-hairpin RNA was generated by GenePharma Company (Suzhou, China). Briefly, two complementary oligonucleotides were synthesized, annealed and cloned into a HIV-based lentiviral expression vector to express a p21-specific short-hairpin. The lentiviral particles were then produced by cotransfecting short-hairpin RNA expression plasmids with packaging plasmids into 293 packaging cells. After 72 h, viruses were collected and the titer was determined. To generate p21-stable knockdown or control cell lines, HeLa cells were infected with the lentiviral particles and selected with puromycin for 3 weeks.

### Generation of p21-KO HEK293T cells

p21-KO HEK293T cells were established using the CRISPR/Cas9 system. The p21 gene target sequences, #1 5′-caccgAGATCAGCCGGCGTTTGGAG-3′ and #2 5′-caccgCCGCGACTGTGATGCGCTAA-3′, were inserted into the guide RNA (gRNA) expression cassette of the pX458-EGFP vector, which also contains an expression cassette of Cas9. The pX458-EGFP plasmid containing the p21 target sequence was then transfected into HEK293T cells. The transfected cells were trypsinized 24 h later into single cells, which were diluted and inoculated into a 96-well plate by flow cytometry. Each colony was individually propagated in a 96-well plate, and the knockout of p21 expression was confirmed by western blotting and PCR.

### Real-time quantitative PCR analysis

The RNA samples from cells were extracted using Trizol reagent (Invitrogen) in accordance with the manufacturer’s instructions. The quality of RNA was verified by evaluating the OD at 260/280 nm. The cDNA was then generated with 1 μg of total RNA using the Superscript III first-strand synthesis SuperMix (Invitrogen) according to the manufacturer’s protocol. For one real-time reaction, 20 mL of SYBR Green PCR reaction mix (Applied Biosystems), including a 1/10 volume of the synthesized cDNA plus an appropriate oligonucleotide primer pair, were analyzed on a LightCycler 480 (Roche). The comparative Ct method was used to determine the relative mRNA expression of genes normalized by the housekeeping gene GAPDH. The specific primers used are shown in S1 Table.

### Polymerase activity assay

A dual-luciferase reporter assay system (Promega) was used to compare the polymerase activities of viral RNP complexes. The PB2, PB1, NP and PA genes from AH1 virus were cloned into the pcDNA3.1 expression plasmid. HEK 293T cells were transfected with PB2, PB1, PA and NP expression plasmids (125 ng of each plasmid), together with the construct pHH21-SC09NS F-Luc (5 ng), and an internal control pRL-TK (10 ng). At 48 h post-transfection, cell lysates were prepared using the dual luciferase reporter assay system (Promega), and the luciferase activities were measured on a GloMax 96 microplate luminometer (Promega). Three independent experiments were performed.

### Confocal microscopy

A549 cells were transfected with expression plasmids for Flag-tagged p21. After 24 h, cells were infected with AH1 virus at a MOI of 1 for 18 h, along with uninfected controls. Cells were fixed in 4% paraformaldehyde (PFA) and permeabilized with 0.2% Triton X-100, then blocked for 60 min at room temperature with 2% bovine serum albumin and 7% fetal bovine serum in PBS. Cells were incubated overnight at 4°C with primary antibodies, followed by Alexa Fluor 594 goat anti-mouse IgG or Alexa Fluor 488 goat anti-rabbit IgG for 1 h. Finally, cover slips were mounted onto microscope slides along with 10–20 μL of DAPI for 3 min and slides were examined by confocal microscopy. Images were processed using a FluoView FV3000 confocal laser scanning microscope (Olympus) and analyzed using the Imaris 9.2 platform.

### Western blot analysis

Cells were lysed in RIPA lysis buffer containing 1 mM phenylmethylsulfonyl fluoride (PMSF), and the total protein was quantitated with a bicinchoninic acid (BCA) protein assay kit (Beyotime, Haimen, China). Proteins from cell extracts (20 to 50 μg) were denatured at 100°C for 10 min, fractionated on a 12% sodium dodecyl sulfate (SDS)-polyacrylamide gel, and subsequently electroblotted onto a polyvinylidene difluoride (PVDF) membrane (GE Healthcare, Pittsburgh, PA, USA). After blocking in 5% non-fat dry milk in Tris-buffered saline with Tween 20 (10 mM Tris-HCl, pH 8.0; 150 mM NaCl; 0.5% Tween 20), membranes were incubated with primary antibodies overnight at 4°C. After washing, the secondary antibody (horseradish peroxidase-conjugated species-specific antisera; 1:10000 dilution) was added and incubated for 1 h, and bound antibody was visualized using an enhanced chemiluminescence system (Thermo Fisher Scientific, Waltham, MA, USA). When indicated, cytosolic proteins were normalized to β-actin.

### Co-immunoprecipitation assay

To examine the interaction of proteins in transfected cells, HEK293T cells were transfected with the indicated plasmids using a jetPRIME kit (Polyplus Transfection, Illkirch, France). After 24 h of transfection, cells were washed with PBS and lysed in 250 μL of RIPA lysis buffer containing freshly added protease and phosphatase inhibitors (Roche Molecular Biochemicals, Rotkreuz, Switzerland). Protein lysates (500 μg) were used for immunoprecipitation and were incubated overnight with anti-Flag or anti-HA magnetic beads (Bimake, Houston, TX, USA). After washing with lysis buffer, proteins were analyzed by western blotting.

### GST pull-down assay

The GST, GST-p21 and GST-HO-1 proteins were obtained using an *E*. *coli* prokaryotic expression system. HEK293T cells grown in 10-cm dishes were individually transfected with 10 μg of each plasmid (pCMV-HA-PA or pCMV-HA-HO-1) using the jetPRIME reagents (Polyplus Transfection, Illkirch, France). At 48 h post-transfection, cells were solubilized with 0.5 mL of IP buffer (Beyotime, Haimen, China). Then, cleared lysates from transfected cells were mixed with GST or GST-p21 protein expressed in *E*. *coli* and Glutathione Sepharose 4 Fast Flow (GE Healthcare, Pittsburgh, PA, USA) and the mixtures were incubated for 1 h at 4°C with agitation. After three washes, the bound proteins were separated by SDS-PAGE. GST-tagged proteins in the eluates were detected by Coomassie blue staining, and non-GST tagged proteins were detected by western blotting.

### Peptide synthesis

The peptide mimic was synthesized by GenScript (Nanjing, China). In short, transmembrane peptide was added to the C-terminus of the peptide, along with PEG modification, to enable it to pass through the cell membrane. The amino acid sequence was as follows: ALMAGCIQ{PEG2}YGRKKRRQRRR.

### Mass spectrometry analysis

Mass spectrometry was performed and the results were analyzed using Applied Protein Technology (Shanghai, China). In brief, 20 μg of protein for each sample were mixed with 5× loading buffer and boiled for 5 min. The proteins were separated by 12.5% SDS-PAGE (constant current 14 mA, 90 min). Protein bands were visualized by Coomassie blue R-250 staining and protein identification was performed by bioinformatic analysis.

### Animal experiments

All animal studies were carried out in strict accordance with the recommendations of the Guide for the Care and Use of Laboratory Animals of the Ministry of Science and Technology of the People’s Republic of China, as well as the institutional guidelines of China Agricultural University (CAU) (approval SKLAB-B-2017-003), and were approved by the Beijing Association for Science and Technology of China (approval SYXK, Beijing, 2007–0023). Groups of 6- to 8-week-old female C57/BL6J mice (Gempharmatech, Nanjing, China) were anesthetized with Zoletil (tiletamine-zolazepam; Virbac; 20 μg/g) and intranasally inoculated with 50 μL of diluted infectious virus in PBS. For AH1 viruses, 50 TCID_50_ (peptide experiment) or 100 TCID_50_ of each test virus was used for inoculation. Six mice from each group were euthanized at 3 and 5 dpi, and viral titers in the lung were determined by the infection of MDCK cells. The body weight and survival of mice were monitored daily from day 1 post-infection.

### Histological analysis

After euthanization, the lung tissues of WT and p21^˗/˗^ mice were removed from the thorax, washed with PBS, and fixed with 4% formaldehyde. After fixation of the lung tissue and processing in paraffin wax, sections (5 μm thick) were cut longitudinally through the left and right lungs and stained with hematoxylin and eosin (H&E) for histopathological assessment. Histopathological scoring was conducted according to a previously published study [51]^.^ In brief, lung microscopic lesions were blindly evaluated from 0 to 4 in five random fields to account for the distribution and severity of interstitial pneumonia.

### Immunohistochemical analysis

The lung tissue sections were quenched in 3% H_2_O_2_ and then pretreated with proteinase K for 15 min. Slides were blocked for 30 min, rinsed with Tris-buffered saline with Tween 20 (TBST) and incubated with a rabbit polyclonal antibody specific for type A influenza nucleoprotein antigen (AA5H; Abcam, Hong Kong) at a 1:100 dilution for 2 h. The sections were then incubated with horseradish peroxidase-conjugated secondary antibody for 60 min at room temperature. The signal was detected using a Vector Elite ABC Kit (Vectastain, Vector Laboratories, Burlingame, CA, USA). The tissue sections were also stained with H&E for routine morphologic analysis.

### Statistical analysis

Values are shown as the mean ± SEM. All experiments were performed with at least three-independent repeats giving consistent results. A Student’s t test was used to compare differences between two groups. One-way ANOVA or two-way ANOVA was performed for multiple comparisons using GraphPad Prism v8.0 (GraphPad Software Inc., San Diego, CA, USA). The Kaplan–Meier method was employed to analyze the survival rates. A *P*-value less than 0.05 was considered statistically different.

## Supporting information

S1 Fig Differentially expressed genes in A549 cells after IAV infection. **(A** and **B)**, Differentially expressed genes in A549 cells after IAV infection. A549 cells were infected with AH1 viruses at a 0.1 MOI for 18 h. Total RNA was extracted and used for RNA-Seq analysis. Results were obtained based on a threshold fold-change of Z > 2.0 and a *P*-value <10^˗90^. (**C**) A549 cells were infected with UV-treated AH1 viruses for different times. For **C**, data are representative of three independent experiments.
(TIF)

S2 Fig p21 inhibits the replication of multiple subtypes of IAV. (**A**) and (**B**) A549 cells were transfected with siRNA oligonucleotides against p21 or shRNA and infected with a 0.1 MOI of AH1 virus. The expression of proteins was detected by western blotting. (**C** and **D**) A549 cells were transfected with scrambled control siRNA and siRNA oligonucleotides against p21 and infected with AH1 virus. Cell viability and growth rate were assessed using a CCK-8 kit and a cell count assay, respectively. (**E**) NHBE cells treated with siControl or siRNA#1 were infected with AH1 virus at 0.01 MOI. Cell lysates were collected and analyzed by western blotting. Supernatants were titrated by a TCID_50_ assay. (**F**) A549 cells were treated with UC2288 (5 μM) or the vehicle control and infected with AH1 virus at 0.1 MOI. Cell lysates were collected and analyzed by western blotting, and the virus yields were determined by a TCID_50_ assay. (**G** to **I**) A549 or HeLa cells treated with Flag-tagged p21 plasmids were infected with AH1 virus at 0.1 MOI. Cell viability and growth rate were assessed using a CCK-8 kit and a cell count assay, respectively. Cell lysates were collected and analyzed by western blotting, and the virus yields were determined by a TCID_50_ assay. (**J**) A549 cells treated with siControl or siRNA#1 were infected with H3, H7 and H1 subtype viruses at 0.1 MOI. Cell lysates were collected and analyzed by western blotting. (**K**) A549 cells treated with siControl or siRNA#1 were infected with AH1 virus at 0.1 MOI. Cell lysates were collected at the indicated time points and analyzed by RT-PCR. All data are representative or presented as the mean ± SEM of three independent experiments unless specified. *P < 0.05, **P < 0.05.
(TIF)

S3 Fig Inhibition of p21 increases the pathogenicity of IAV in mice. (**A**) C57BL/6J mice were orally gavaged with UC2288 or corn oil (n = 6 mice in each group). After 24 h, the mice were euthanized and the lungs were subjected to immunoblotting. (**B**) WT or p21^-/-^ mice were infected with AH1 virus. Cytokines were detected using a CBA kit at 5 dpi. (**C**) Immunohistochemically stained sections of the mouse lungs infected with AH1 viruses demonstrating the presence of IAV. Scale bar = 100 μm. All data are representative or presented as the mean ± SEM of three independent experiments unless specified.*P < 0.05.
(TIF)

S4 Fig Identification of IAV proteins that interact with p21. (**A**) Co-IP assay of HA-NP, HA-PB2, HA-PB1, HA-M1, HA-NS1 and Flag-p21 in HEK293T cells. HEK293T cells were transfected individually or in combination with plasmids that expressed Flag-p21, HA-NP, HA-PB2, HA-PB1, HA-M1 or HA-NS1. Cell lysates were immunoprecipitated with anti-Flag mAb and were subjected to western blotting. (**B** and **C**) p53-knockout HCT116 cells or IFNR-knockout A549 cells were infected with AH1 virus and whole cell lysates were collected and analyzed by western blotting. (**D**) A549 cells were transfected with different amounts of PB2 expression vectors and the protein levels of p21 were detected. All data are representative of three independent experiments.
(TIF)

S5 Fig The genotyping results of WT and p21-KO HEK293T cells. (**A-D**) Generation of p21-KO HEK293T cells. Genomic DNA was extracted and purified from HEK293T cells using the DNeasy Blood and Tissue Kit (Qiagen). PCR and sequencing were performed to identify the WT and KO cells. NC, ddH_2_O was used as a negative control. The genotype of the generated HEK293T cells was identified by western blotting. (**E**) HA-PB1, PB2 and p21 were co-transfected into p21-KO HEK293 cells. After 48 h, cell lysates were harvested for immunoprecipitation using anti-HA antibody and blotted using the indicated antibodies. For **D** and **E**, data are representative of three independent experiments.
(TIF)

S6 Fig Differentially expressed gene analysis in the WT and p21-KO A549 cells after IAV infection. KEGG analysis and differentially expressed genes in the WT and p21-KO cell lines after virus infection. A549 cells were transfected with scrambled control siRNA and siRNA oligonucleotides against p21. After 24 h, the cells were infected with a 0.1 MOI of AH1. Total RNA was extracted and used for RNA-Seq analysis.
(TIF)

S7 Fig p21 inhibits ubiquitin degradation of HO-1 protein. (**A**) WT or p21-KO HEK293T cells were pretreated with 25 mM CHX and incubated for the time periods indicated. Endogenous HO-1 was detected, and the intensity of the HO-1 bands was quantified and plotted on a semi-log graph. (**B**) HEK293T cells treated with p21 expression vectors were pretreated with 25 mM CHX. Endogenous HO-1 was detected for the time periods indicated. (**C**) WT and p21-KO HEK293T cells were treated with 10 mM of MG132. Cell lysates were subjected to an *in vivo* ubiquitination assay for the detection of the ubiquitin-conjugated endogenous HO-1 protein. (**D**) A549 cells were transfected with HO-1 plasma or siHO-1 oligonucleotides and infected with AH1 virus. Cell lysates were collected and analyzed by RT-qPCR. (**E**) and (**F**) p21^˗/˗^ mice were orally gavaged with hemin or an equal volume of vehicle control every other day and challenged with AH1 virus. Lung tissues were collected and analyzed by western blotting and immunohistochemistry. Scale bar = 100 μm. For **A–C** and **E–F**, data are representative of three independent experiments. For **A, B** and **D,** data are presented as the mean ± SEM from three independent experiments. *P < 0.05, **P < 0.05.
(TIF)

S8 Fig p21 peptide mimics inhibit IAV replication. (**A** and **B**) A549 cells were treated with scrambled control peptides or peptide mimics. The cell cycle phase and growth rate were detected by flow cytometry and a CCK-8 kit, respectively. (**C**) C57/BL6J mice were intraperitoneally injected with p21 peptide mimics or scrambled peptides every other day and challenged with AH1 virus at 50 TCID_50_ (n = 6 mice in each group). Lung tissues were collected and analyzed by immunohistochemistry. Scale bar = 100 μm. All data are representative or presented as the mean ± SEM from three independent experiments unless specified.
(TIF)

S9 Fig Enlarging version of S1B Fig. A549 cells were infected with AH1 viruses at a 0.1 MOI for 18 h. Total RNA was extracted and used for RNA-Seq analysis. Results were obtained based on a threshold fold-change of Z > 2.0 and a *P*-value <10^˗90^.
(EMF)

S1 Table Primers used in this study. (DOCX)

S2 Table Host proteins interacting with p21 protein in A549 cells tested by mass spectrometry. (XLSX)

## References

[1] Taubenberger JK, Morens DM. Influenza: The once and future pandemic. Public Health Reports. 2010;125: 15–26. doi: 10.1177/00333549101250s305 20568566 PMC2862331

[2] Fukuyama S, Kawaoka Y. 1-s2.0-S0952791511000896-main.pdf. Current opinion in immunology. 2011;23: 481–486. doi: 10.1016/j.coi.2011.07.016 21840185 PMC3163725

[3] Tscherne DM, García-Sastre A. Virulence determinants of pandemic influenza viruses. Journal of Clinical Investigation. 2011;121: 6–13. doi: 10.1172/JCI44947 21206092 PMC3007163

[4] Higginson R, Davies K. The threat of an avian influenza pandemic. British journal of nursing (Mark Allen Publishing). 2005;14: 632. doi: 10.12968/bjon.2005.14.12.18281 16010211

[5] Morens DM, Taubenberger JK, Harvey HA, Memoli MJ. The 1918 influenza pandemic: Lessons for 2009 and the future. Critical Care Medicine. 2010;38: e10–e20. doi: 10.1097/CCM.0b013e3181ceb25b 20048675 PMC3180813

[6] Allen JD, Ross TM. H3N2 influenza viruses in humans: Viral mechanisms, evolution, and evaluation. Human Vaccines and Immunotherapeutics. 2018;14: 1840–1847. doi: 10.1080/21645515.2018.1462639 29641358 PMC6149781

[7] Choi WS, Lloren KKS, Baek YH, Song MS. The significance of avian influenza virus mouse-adaptation and its application in characterizing the efficacy of new vaccines and therapeutic agents. Clinical and Experimental Vaccine Research. 2017/07/26. 2017;6: 83–94. doi: 10.7774/cevr.2017.6.2.83 28775972 PMC5540968

[8] Hussain M, Galvin HD, Haw TY, Nutsford AN, Husain M. Drug resistance in influenza a virus: The epidemiology and management. Infection and Drug Resistance. 2017;10: 121–134. doi: 10.2147/IDR.S105473 28458567 PMC5404498

[9] Watanabe T, Kawakami E, Shoemaker JE, Lopes TJS, Matsuoka Y, Tomita Y, et al. Influenza virus-host interactome screen as a platform for antiviral drug development. Cell Host and Microbe. 2014;16: 795–805. doi: 10.1016/j.chom.2014.11.002 25464832 PMC4451456

[10] Jefferson T, Jones MA, Doshi P, Del Mar CB, Hama R, Thompson M, et al. Neuraminidase inhibitors for preventing and treating influenza in healthy adults and children. Sao Paulo Medical Journal. 2014;132: 256–257. doi: 10.1590/1516-3180.20141324t2 25055075 PMC10496739

[11] Murti KG, Webster RG, Jones IM. Localization of RNA polymerases on influenza viral ribonucleoproteins by immunogold labeling. Virology. 1988;164: 562–566. doi: 10.1016/0042-6822(88)90574-0 3369093

[12] Klumpp K, Ruigrok RWH, Baudin F. Roles of the influenza virus polymerase and nucleoprotein in forming a functional RNP structure. EMBO Journal. 1997;16: 1248–1257. doi: 10.1093/emboj/16.6.1248 9135141 PMC1169723

[13] Neumann G, Brownlee GG, Fodor E, Kawaoka Y. Orthomyxovirus replication, transcription, and polyadenylation. Current Topics in Microbiology and Immunology. 2004;283: 121–143. doi: 10.1007/978-3-662-06099-5_4 15298169

[14] Huang TS, Palese P, Krystal M. Determination of influenza virus proteins required for genome replication. Journal of Virology. 1990;64: 5669–5673. doi: 10.1128/JVI.64.11.5669-5673.1990 2214032 PMC248627

[15] Noda T, Kawaoka Y. Structure of influenza virus ribonucleoprotein complexes and their packaging into virions. Reviews in Medical Virology. 2010;20: 380–391. doi: 10.1002/rmv.666 20853340 PMC6029254

[16] Coloma R, Valpuesta JM, Arranz R, Carrascosa JL, Ortín J, Martín-Benito J. The structure of a biologically active influenza virus ribonucleoprotein complex. PLoS Pathogens. 2009;5: e1000491. doi: 10.1371/journal.ppat.1000491 19557158 PMC2695768

[17] Stubbs TM, Te Velthuis AJW. The RNA-dependent RNA polymerase of the influenza A virus. Future virology. 2014;9: 863–876. doi: 10.2217/fvl.14.66 25431616 PMC4243023

[18] Chen X, Liu S, Goraya MU, Maarouf M, Huang S, Chen J-L. Host immune response to influenza A virus infection. Frontiers in immunology. 2018;9: 320. doi: 10.3389/fimmu.2018.00320 29556226 PMC5845129

[19] Katze MG, He Y, Gale M. Viruses and interferon: a fight for supremacy. Nature Reviews Immunology. 2002;2: 675–687. doi: 10.1038/nri888 12209136

[20] Tzima S, Victoratos P, Kranidioti K, Alexiou M, Kollias G. Myeloid heme oxygenase-1 regulates innate immunity and autoimmunity by modulating IFN-β production. Journal of Experimental Medicine. 2009;206: 1167–1179. doi: 10.1084/jem.20081582 19398754 PMC2715044

[21] Balomenos D, Martín-Caballero J, García MI, Prieto I, Flores JM, Serrano M, et al. The cell cycle inhibitor p21 controls T-cell proliferation and sex- linked lupus development. Nature Medicine. 2000;6: 171–176. doi: 10.1038/72272 10655105

[22] Salvador JM, Hollander MC, Nguyen AT, Kopp JB, Barisoni L, Moore JK, et al. Mice lacking the p53-effector gene Gadd45a develop a lupus-like syndrome. Immunity. 2002;16: 499–508. doi: 10.1016/s1074-7613(02)00302-3 11970874

[23] Zhu B, Symonds ALJ, Martin JE, Kioussis D, Wraith DC, Li S, et al. Early growth response gene 2 (Egr-2) controls the self-tolerance of T cells and prevents the development of lupuslike autoimmune disease. Journal of Experimental Medicine. 2008;205: 2295–2307. doi: 10.1084/jem.20080187 18779345 PMC2556781

[24] Chen H, Li C, Huang J, Cung T, Seiss K, Beamon J, et al. CD4 + T cells from elite controllers resist HIV-1 infection by selective upregulation of p21. Journal of Clinical Investigation. 2011;121: 1549–1560. doi: 10.1172/JCI44539 21403397 PMC3069774

[25] Lloberas J, Celada A. P21waf1/CIP1, a CDK inhibitor and a negative feedback system that controls macrophage activation. European Journal of Immunology. 2009;39: 691–694. doi: 10.1002/eji.200939262 19283709

[26] Laphanuwat P, Jirawatnotai S. Immunomodulatory Roles of Cell Cycle Regulators. Frontiers in Cell and Developmental Biology. 2019;7: 23. doi: 10.3389/fcell.2019.00023 30863749 PMC6399147

[27] Rackov G, Hernández-Jiménez E, Shokri R, Carmona-Rodríguez L, Mañes S, Álvarez-Mon M, et al. P21 mediates macrophage reprogramming through regulation of p50-p50 NF-κB and IFN-β. Journal of Clinical Investigation. 2016;126: 3089–3103. doi: 10.1172/JCI83404 27427981 PMC4966310

[28] Kihara S, Hayashi S, Hashimoto S, Kanzaki N, Takayama K, Matsumoto T, et al. Cyclin-Dependent Kinase Inhibitor-1-Deficient Mice are Susceptible to Osteoarthritis Associated with Enhanced Inflammation. Journal of Bone and Mineral Research. 2017;32: 991–1001. doi: 10.1002/jbmr.3080 28128866

[29] Trakala M, Arias CF, García MI, Moreno-Ortiz MC, Tsilingiri K, Fernández PJ, et al. Regulation of macrophage activation and septic shock susceptibility via p21 (WAF1/CIP1). European Journal of Immunology. 2009;39: 810–819. doi: 10.1002/eji.200838676 19224635

[30] Valle-Casuso JC, Allouch A, David A, Lenzi GM, Studdard L, Barré-Sinoussi F, et al. p21 Restricts HIV-1 in Monocyte-Derived Dendritic Cells through the Reduction of Deoxynucleoside Triphosphate Biosynthesis and Regulation of SAMHD1 Antiviral Activity. Journal of Virology. 2017;91: 1–18. doi: 10.1128/JVI.01324-17 28931685 PMC5686764

[31] Vázquez N, Greenwell-Wild T, Marinos NJ, Swaim WD, Nares S, Ott DE, et al. Human Immunodeficiency Virus Type 1-Induced Macrophage Gene Expression Includes the p21 Gene, a Target for Viral Regulation. Journal of Virology. 2005;79: 4479–4491. doi: 10.1128/JVI.79.7.4479-4491.2005 15767448 PMC1061522

[32] Bergamaschi A, David A, Le Rouzic E, Nisole S, Barré-Sinoussi F, Pancino G. The CDK Inhibitor p21 Cip1/WAF1 Is Induced by FcγR Activation and Restricts the Replication of Human Immunodeficiency Virus Type 1 and Related Primate Lentiviruses in Human Macrophages. Journal of Virology. 2009;83: 12253–12265. doi: 10.1128/JVI.01395-09 19759136 PMC2786717

[33] Boivin S, Cusack S, Ruigrok RWH, Hart DJ. Influenza A virus polymerase: Structural insights into replication and host adaptation mechanisms. Journal of Biological Chemistry. 2010;285: 28411–28417. doi: 10.1074/jbc.R110.117531 20538599 PMC2937865

[34] Deng T, Engelhardt OG, Thomas B, Akoulitchev A V, Brownlee GG, Fodor E. Role of ran binding protein 5 in nuclear import and assembly of the influenza virus RNA polymerase complex. Journal of virology. 2006;80: 11911–11919. doi: 10.1128/JVI.01565-06 17005651 PMC1676300

[35] Te Velthuis AJW, Fodor E. Influenza virus RNA polymerase: Insights into the mechanisms of viral RNA synthesis. Nature Reviews Microbiology. 2016/07/11. 2016;14: 479–493. doi: 10.1038/nrmicro.2016.87 27396566 PMC4966622

[36] Mogensen TH. Pathogen recognition and inflammatory signaling in innate immune defenses. Clinical Microbiology Reviews. 2009;22: 240–273. doi: 10.1128/CMR.00046-08 19366914 PMC2668232

[37] Takeuchi O, Akira S. Pattern Recognition Receptors and Inflammation. Cell. 2010;140: 805–820. doi: 10.1016/j.cell.2010.01.022 20303872

[38] Yanai H, Chiba S, Hangai S, Kometani K, Inoue A, Kimura Y, et al. Revisiting the role of IRF3 in inflammation and immunity by conditional and specifically targeted gene ablation in mice. Proceedings of the National Academy of Sciences of the United States of America. 2018;115: 5253–5258. doi: 10.1073/pnas.1803936115 29712834 PMC5960330

[39] Cummins NW, Weaver EA, May SM, Croatt AJ, Foreman O, Kennedy RB, et al. Heme oxygenase-1 regulates the immune response to influenza virus infection and vaccination in aged mice. The FASEB Journal. 2012;26: 2911–2918. doi: 10.1096/fj.11-190017 22490782 PMC3382093

[40] Zhao M, Wang L, Li S. Influenza A virus-host protein interactions control viral pathogenesis. International Journal of Molecular Sciences. 2017;18: 1–15. doi: 10.3390/ijms18081673 28763020 PMC5578063

[41] Han J, Perez JT, Chen C, Li Y, Benitez A, Kandasamy M, et al. Genome-wide CRISPR/Cas9 Screen Identifies Host Factors Essential for Influenza Virus Replication. Cell Reports. 2018;23: 596–607. doi: 10.1016/j.celrep.2018.03.045 29642015 PMC5939577

[42] Meier R, Franceschini A, Horvath P, Tetard M, Mancini R, Von Mering C, et al. Genome-wide small interfering RNA screens reveal VAMP3 as a novel host factor required for Uukuniemi virus late penetration. Journal of virology. 2014;88: 8565–8578. doi: 10.1128/JVI.00388-14 24850728 PMC4135934

[43] Hao L, Sakurai A, Watanabe T, Sorensen E, Nidom CA, Newton MA, et al. Drosophila RNAi screen identifies host genes important for influenza virus replication. Nature. 2008;454: 890–893. doi: 10.1038/nature07151 18615016 PMC2574945

[44] Li B, Clohisey SM, Chia BS, Wang B, Cui A, Eisenhaure T, et al. Genome-wide CRISPR screen identifies host dependency factors for influenza A virus infection. Nature communications. 2020;11: 1–18. doi: 10.1038/s41467-019-13993-7 31919360 PMC6952391

[45] Zhou Z, Cao M, Guo Y, Zhao L, Wang J, Jia X, et al. Fragile X mental retardation protein stimulates ribonucleoprotein assembly of influenza A virus. Nature communications. 2014;5: 3259. doi: 10.1038/ncomms4259 24514761

[46] Karlas A, MacHuy N, Shin Y, Pleissner KP, Artarini A, Heuer D, et al. Genome-wide RNAi screen identifies human host factors crucial for influenza virus replication. Nature. 2010;463: 818–822. doi: 10.1038/nature08760 20081832

[47] Al Bitar S, Gali-Muhtasib H. The role of the cyclin dependent kinase inhibitor p21cip1/waf1 in targeting cancer: Molecular mechanisms and novel therapeutics. Cancers. 2019;11: 1475. doi: 10.3390/cancers11101475 31575057 PMC6826572

[48] Fu B, Wang L, Ding H, Schwamborn JC, Li S, Dorf ME. TRIM32 Senses and Restricts Influenza A Virus by Ubiquitination of PB1 Polymerase. PLoS Pathogens. 2015;11: 1–23. doi: 10.1371/journal.ppat.1004960 26057645 PMC4461266

[49] Sun N, Jiang L, Ye M, Wang Y, Wang G, Wan X, et al. TRIM35 mediates protection against influenza infection by activating TRAF3 and degrading viral PB2. Protein and Cell. 2020;11: 894–914. doi: 10.1007/s13238-020-00734-6 32562145 PMC7719147

[50] Luo W, Zhang J, Liang L, Wang G, Li Q, Zhu P, et al. Phospholipid scramblase 1 interacts with influenza A virus NP, impairing its nuclear import and thereby suppressing virus replication. PLoS Pathogens. 2018;14: 1–26. doi: 10.1371/journal.ppat.1006851 29352288 PMC5792031

[51] Wu W, Zhang W, Duggan ES, Booth JL, Zou MH, Metcalf JP. RIG-I and TLR3 are both required for maximum interferon induction by influenza virus in human lung alveolar epithelial cells. Virology. 2015/04/11. 2015;482: 181–188. doi: 10.1016/j.virol.2015.03.048 25880109 PMC4461467

