# Supplementary figures and images for: p21 restricts influenza A virus by perturbing the viral polymerase complex and upregulating type I interferon signaling
Source: PLoS Pathog. 2022 Feb 18;18(2):e1010295. doi: 10.1371/journal.ppat.1010295 (PMC8920271; doi:10.1371/journal.ppat.1010295)

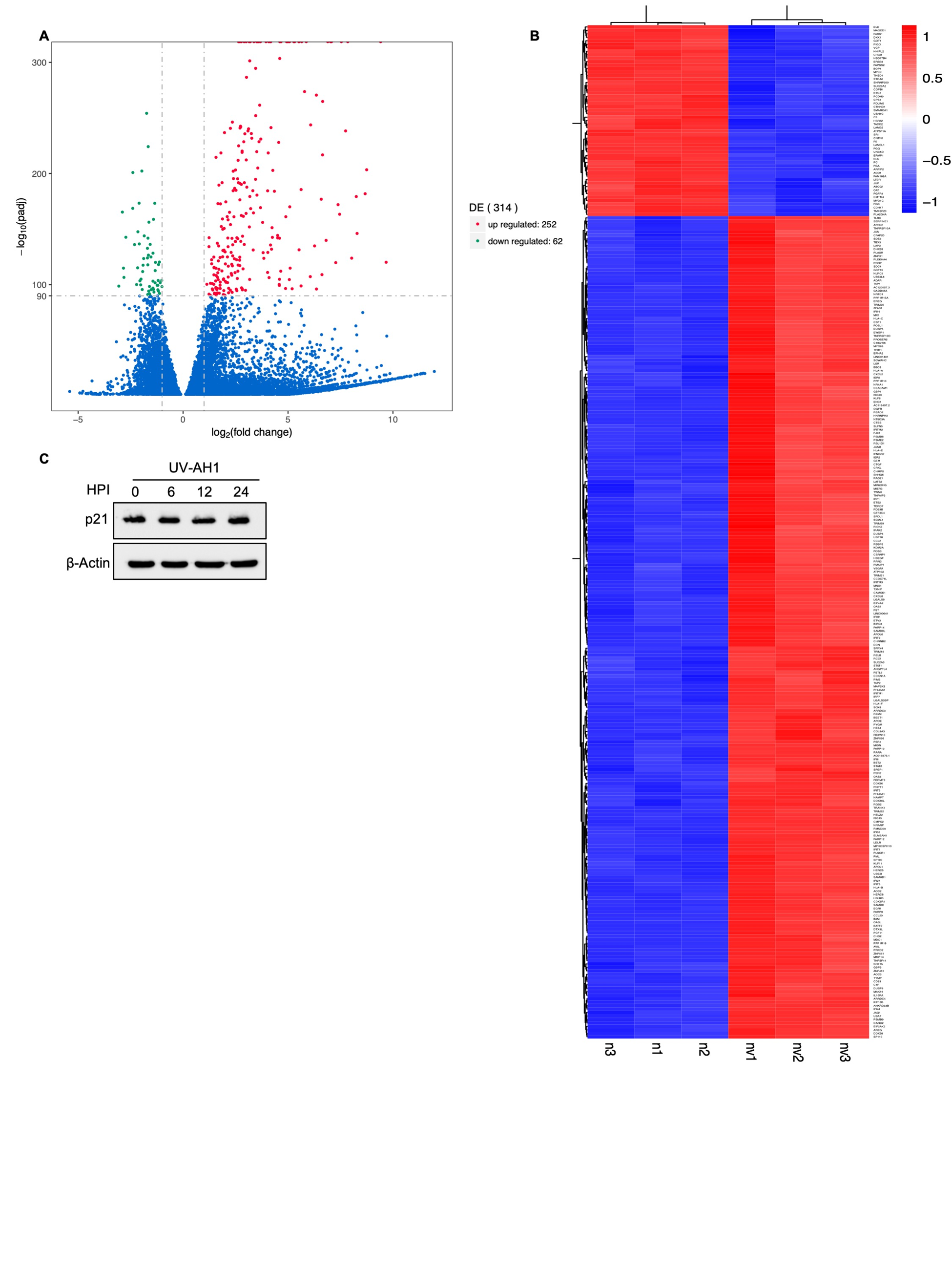

Supplement: S1 Fig — (A and B), Differentially expressed genes in A549 cells after IAV infection. A549 cells were infected with AH1 viruses at a 0.1 MOI for 18 h. Total RNA was extracted and used for RNA-Seq analysis. Results were obtained based on a threshold fold-change of Z > 2.0 and a P-value <10˗90. (C) A549 cells were infected with UV-treated AH1 viruses for different times. For C, data are representative of three independent experiments. (TIF) [file ppat.1010295.s001.tif]

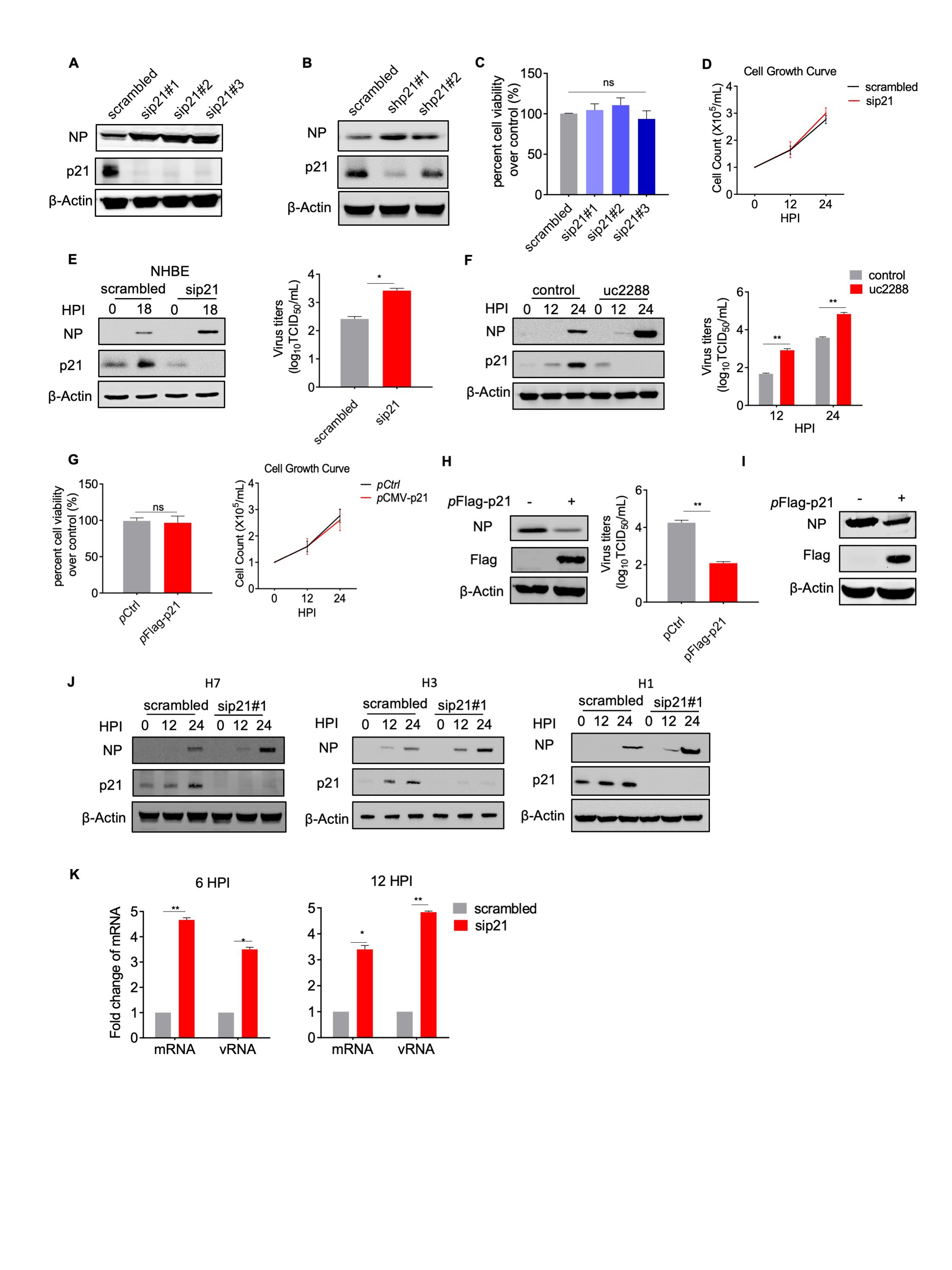

Supplement: S2 Fig — (A) and (B) A549 cells were transfected with siRNA oligonucleotides against p21 or shRNA and infected with a 0.1 MOI of AH1 virus. The expression of proteins was detected by western blotting. (C and D) A549 cells were transfected with scrambled control siRNA and siRNA oligonucleotides against p21 and infected with AH1 virus. Cell viability and growth rate were assessed using a CCK-8 kit and a cell count assay, respectively. (E) NHBE cells treated with siControl or siRNA#1 were infected with AH1 virus at 0.01 MOI. Cell lysates were collected and analyzed by western blotting. Supernatants were titrated by a TCID50 assay. (F) A549 cells were treated with UC2288 (5 μM) or the vehicle control and infected with AH1 virus at 0.1 MOI. Cell lysates were collected and analyzed by western blotting, and the virus yields were determined by a TCID50 assay. (G to I) A549 or HeLa cells treated with Flag-tagged p21 plasmids were infected with AH1 virus at 0.1 MOI. Cell viability and growth rate were assessed using a CCK-8 kit and a cell count assay, respectively. Cell lysates were collected and analyzed by western blotting, and the virus yields were determined by a TCID50 assay. (J) A549 cells treated with siControl or siRNA#1 were infected with H3, H7 and H1 subtype viruses at 0.1 MOI. Cell lysates were collected and analyzed by western blotting. (K) A549 cells treated with siControl or siRNA#1 were infected with AH1 virus at 0.1 MOI. Cell lysates were collected at the indicated time points and analyzed by RT-PCR. All data are representative or presented as the mean ± SEM of three independent experiments unless specified. *P < 0.05, **P < 0.05. (TIF) [file ppat.1010295.s002.tif]

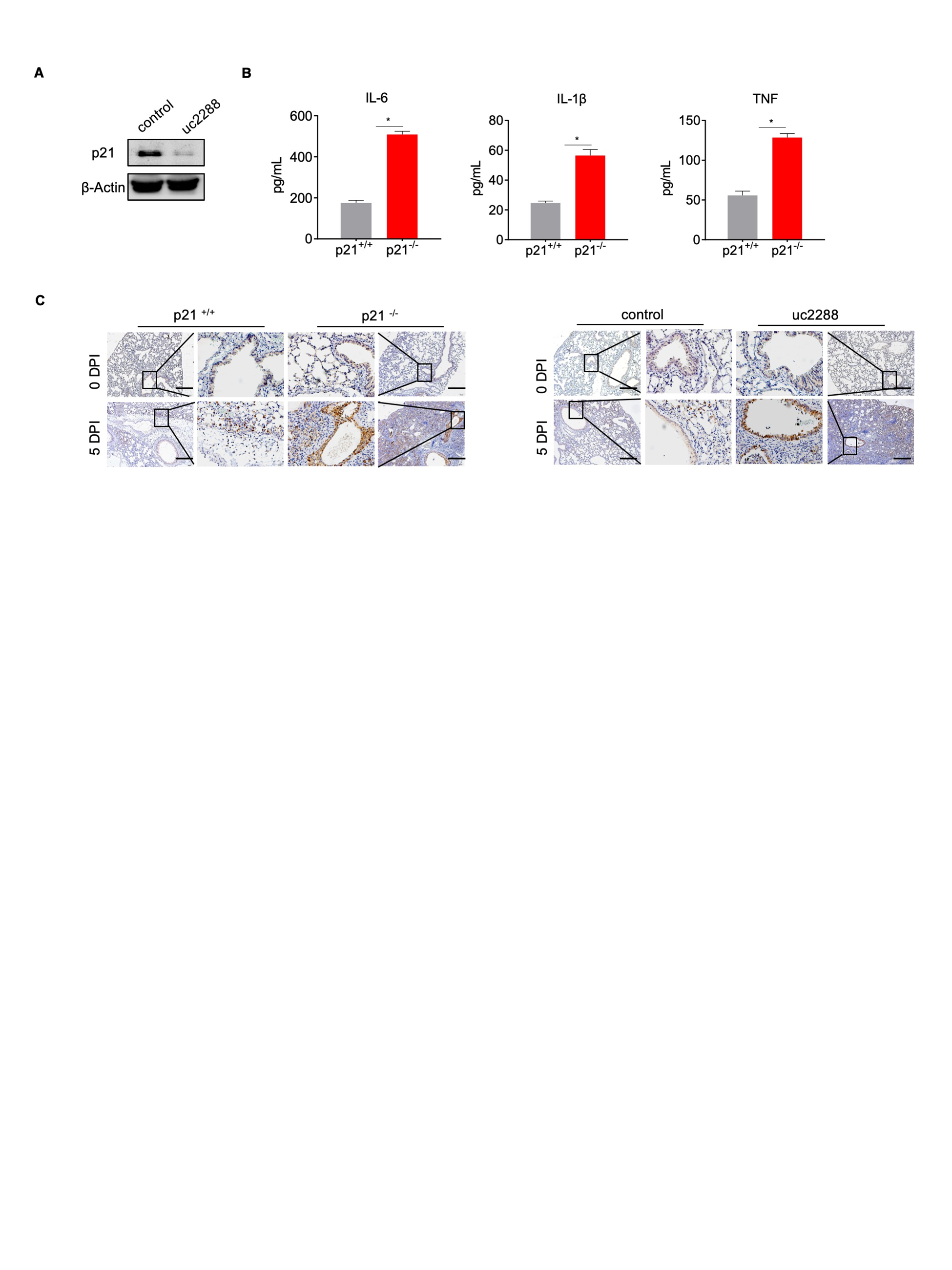

Supplement: S3 Fig — (A) C57BL/6J mice were orally gavaged with UC2288 or corn oil (n = 6 mice in each group). After 24 h, the mice were euthanized and the lungs were subjected to immunoblotting. (B) WT or p21-/- mice were infected with AH1 virus. Cytokines were detected using a CBA kit at 5 dpi. (C) Immunohistochemically stained sections of the mouse lungs infected with AH1 viruses demonstrating the presence of IAV. Scale bar = 100 μm. All data are representative or presented as the mean ± SEM of three independent experiments unless specified.*P < 0.05. (TIF) [file ppat.1010295.s003.tif]

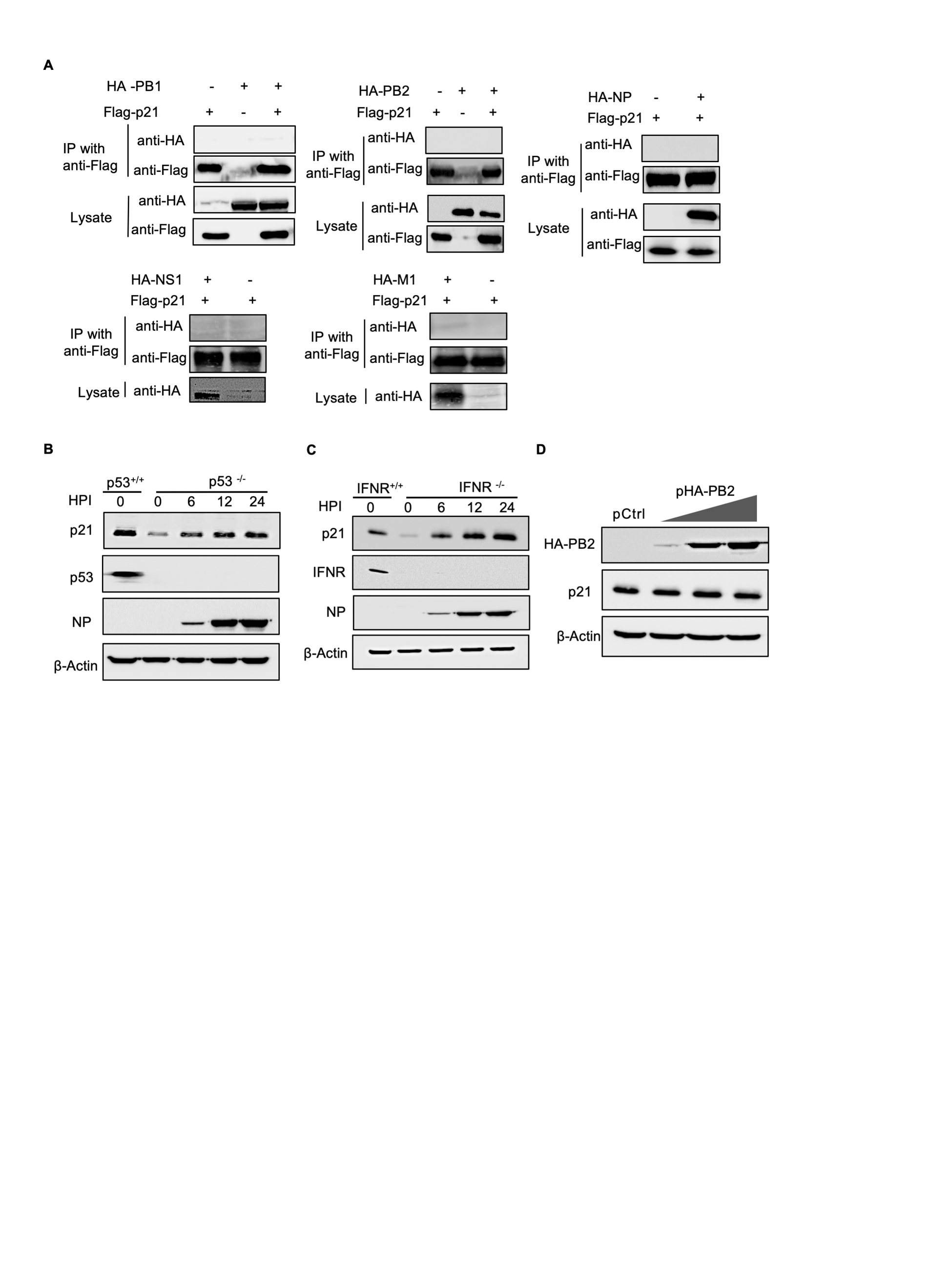

Supplement: S4 Fig — (A) Co-IP assay of HA-NP, HA-PB2, HA-PB1, HA-M1, HA-NS1 and Flag-p21 in HEK293T cells. HEK293T cells were transfected individually or in combination with plasmids that expressed Flag-p21, HA-NP, HA-PB2, HA-PB1, HA-M1 or HA-NS1. Cell lysates were immunoprecipitated with anti-Flag mAb and were subjected to western blotting. (B and C) p53-knockout HCT116 cells or IFNR-knockout A549 cells were infected with AH1 virus and whole cell lysates were collected and analyzed by western blotting. (D) A549 cells were transfected with different amounts of PB2 expression vectors and the protein levels of p21 were detected. All data are representative of three independent experiments. (TIF) [file ppat.1010295.s004.tif]

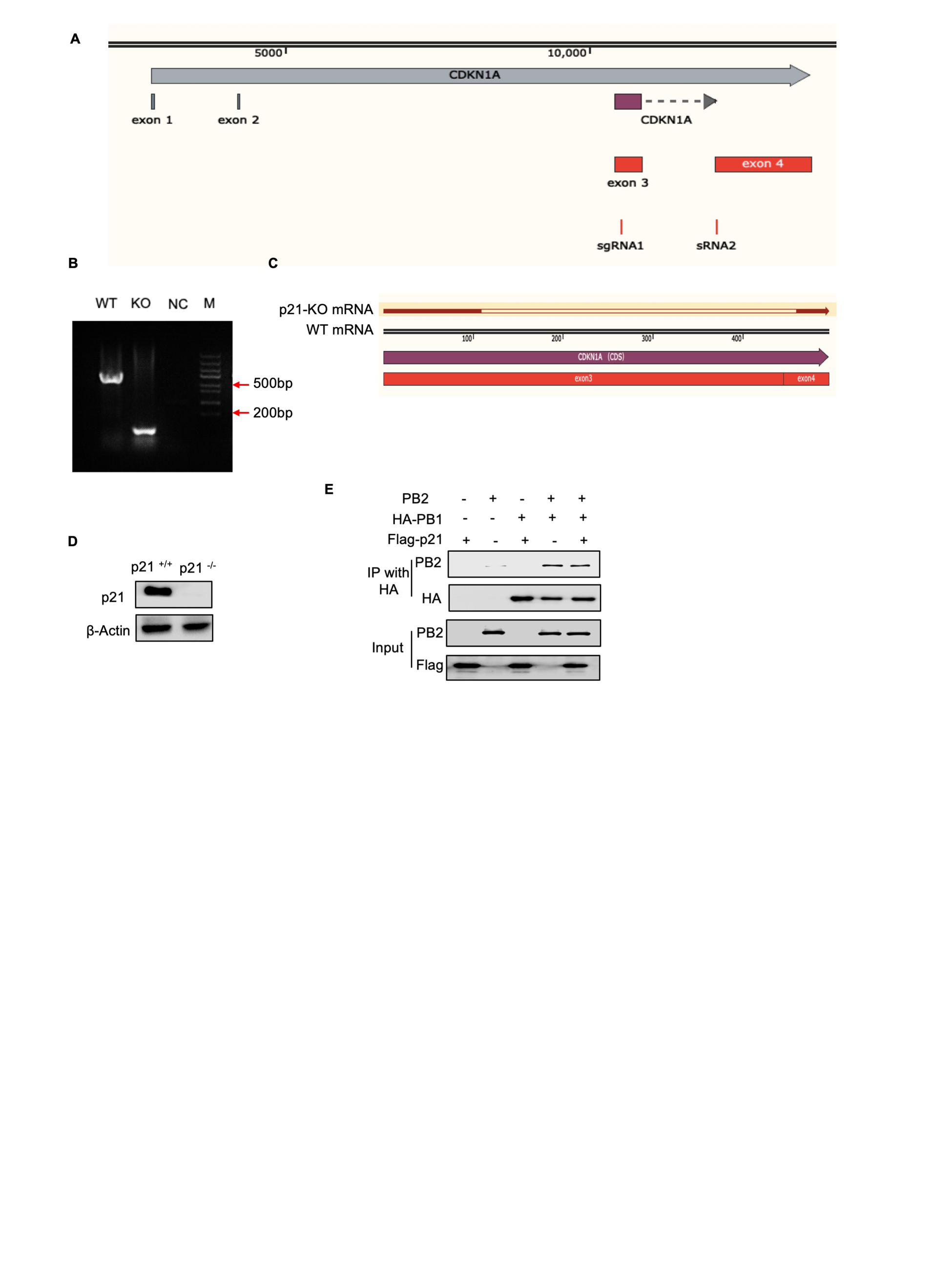

Supplement: S5 Fig — (A-D) Generation of p21-KO HEK293T cells. Genomic DNA was extracted and purified from HEK293T cells using the DNeasy Blood and Tissue Kit (Qiagen). PCR and sequencing were performed to identify the WT and KO cells. NC, ddH2O was used as a negative control. The genotype of the generated HEK293T cells was identified by western blotting. (E) HA-PB1, PB2 and p21 were co-transfected into p21-KO HEK293 cells. After 48 h, cell lysates were harvested for immunoprecipitation using anti-HA antibody and blotted using the indicated antibodies. For D and E, data are representative of three independent experiments. (TIF) [file ppat.1010295.s005.tif]

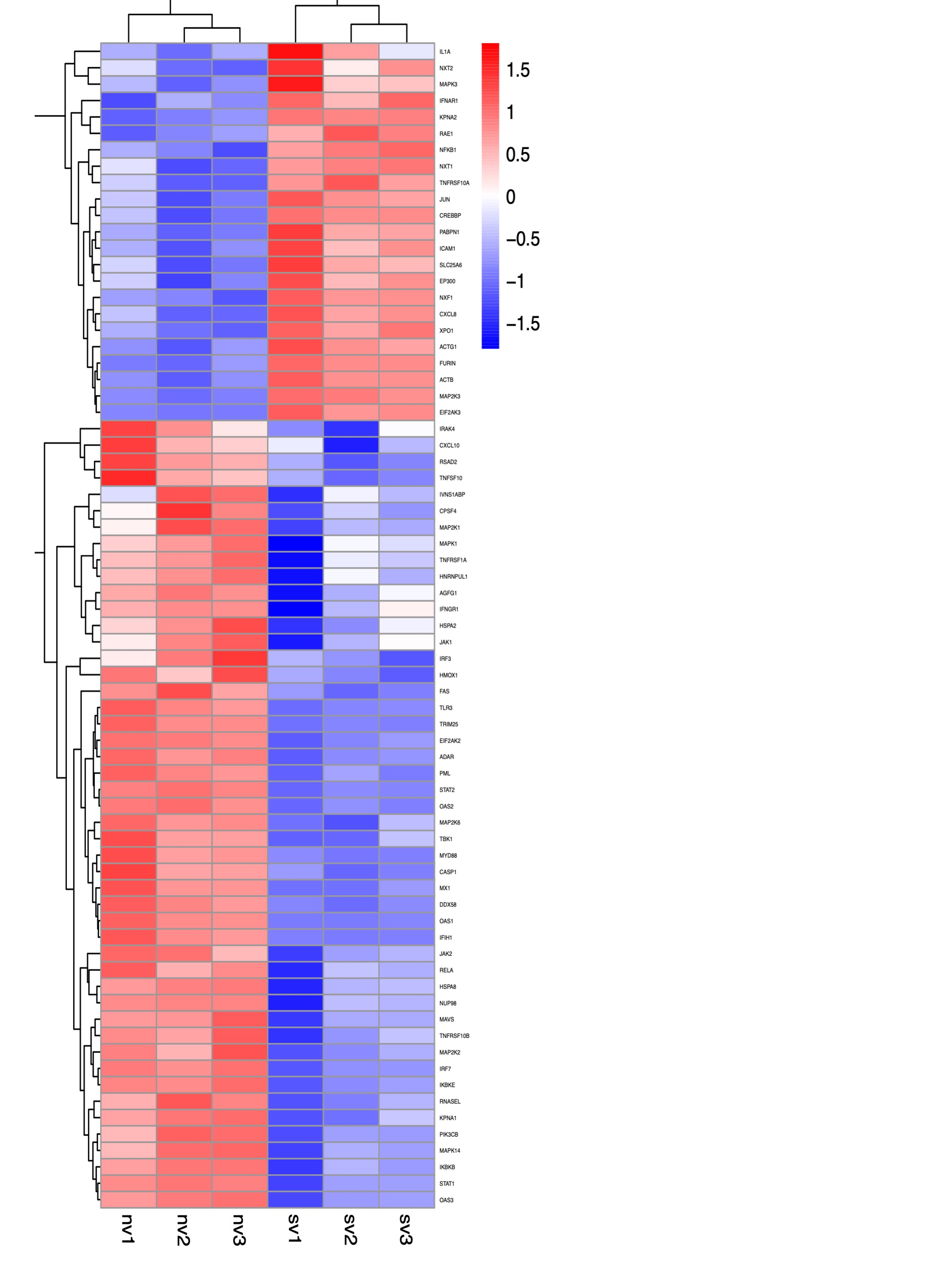

Supplement: S6 Fig — KEGG analysis and differentially expressed genes in the WT and p21-KO cell lines after virus infection. A549 cells were transfected with scrambled control siRNA and siRNA oligonucleotides against p21. After 24 h, the cells were infected with a 0.1 MOI of AH1. Total RNA was extracted and used for RNA-Seq analysis. (TIF) [file ppat.1010295.s006.tif]

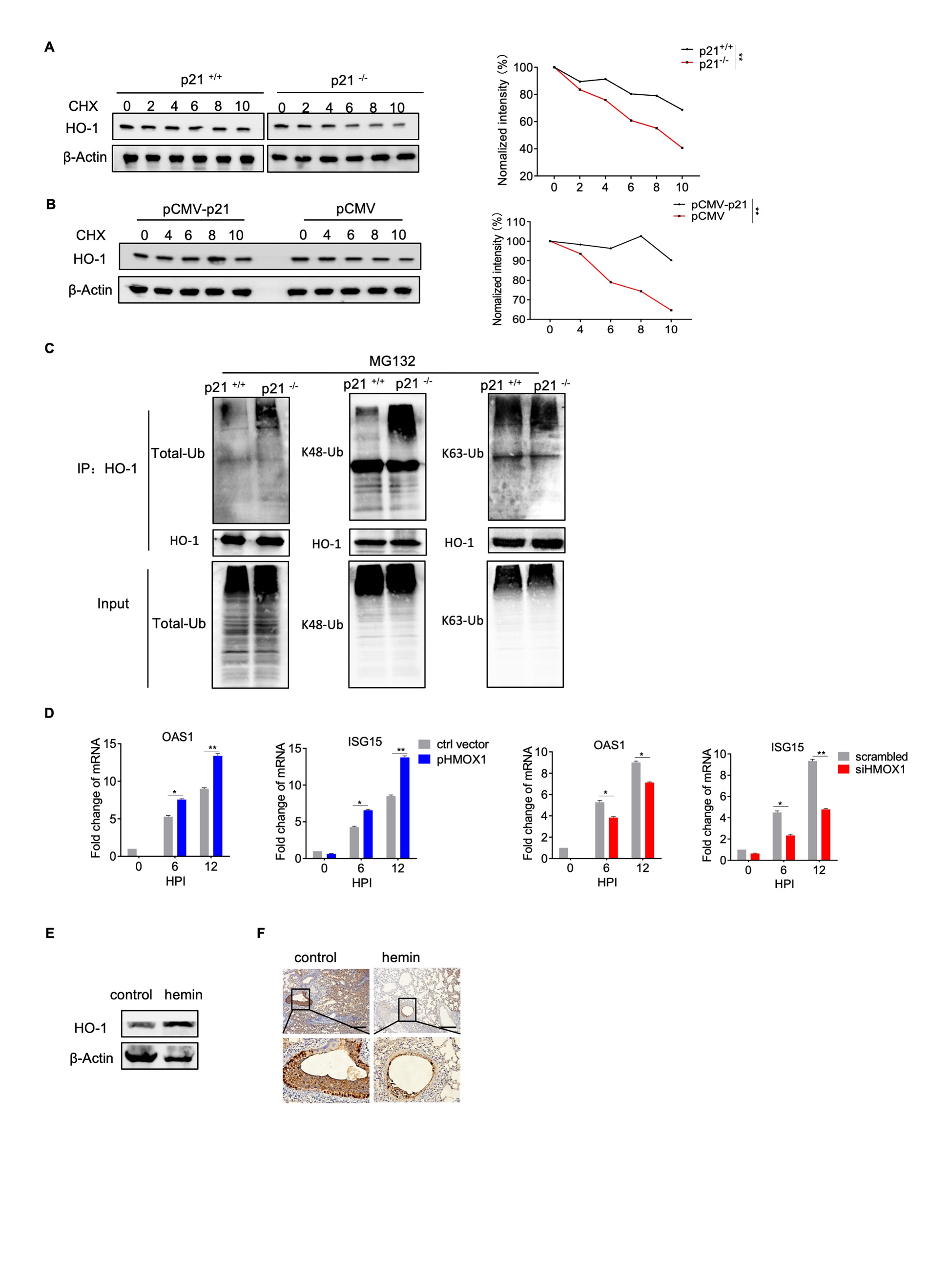

Supplement: S7 Fig — (A) WT or p21-KO HEK293T cells were pretreated with 25 mM CHX and incubated for the time periods indicated. Endogenous HO-1 was detected, and the intensity of the HO-1 bands was quantified and plotted on a semi-log graph. (B) HEK293T cells treated with p21 expression vectors were pretreated with 25 mM CHX. Endogenous HO-1 was detected for the time periods indicated. (C) WT and p21-KO HEK293T cells were treated with 10 mM of MG132. Cell lysates were subjected to an in vivo ubiquitination assay for the detection of the ubiquitin-conjugated endogenous HO-1 protein. (D) A549 cells were transfected with HO-1 plasma or siHO-1 oligonucleotides and infected with AH1 virus. Cell lysates were collected and analyzed by RT-qPCR. (E) and (F) p21˗/˗ mice were orally gavaged with hemin or an equal volume of vehicle control every other day and challenged with AH1 virus. Lung tissues were collected and analyzed by western blotting and immunohistochemistry. Scale bar = 100 μm. For A–C and E–F, data are representative of three independent experiments. For A, B and D, data are presented as the mean ± SEM from three independent experiments. *P < 0.05, **P < 0.05. (TIF) [file ppat.1010295.s007.tif]

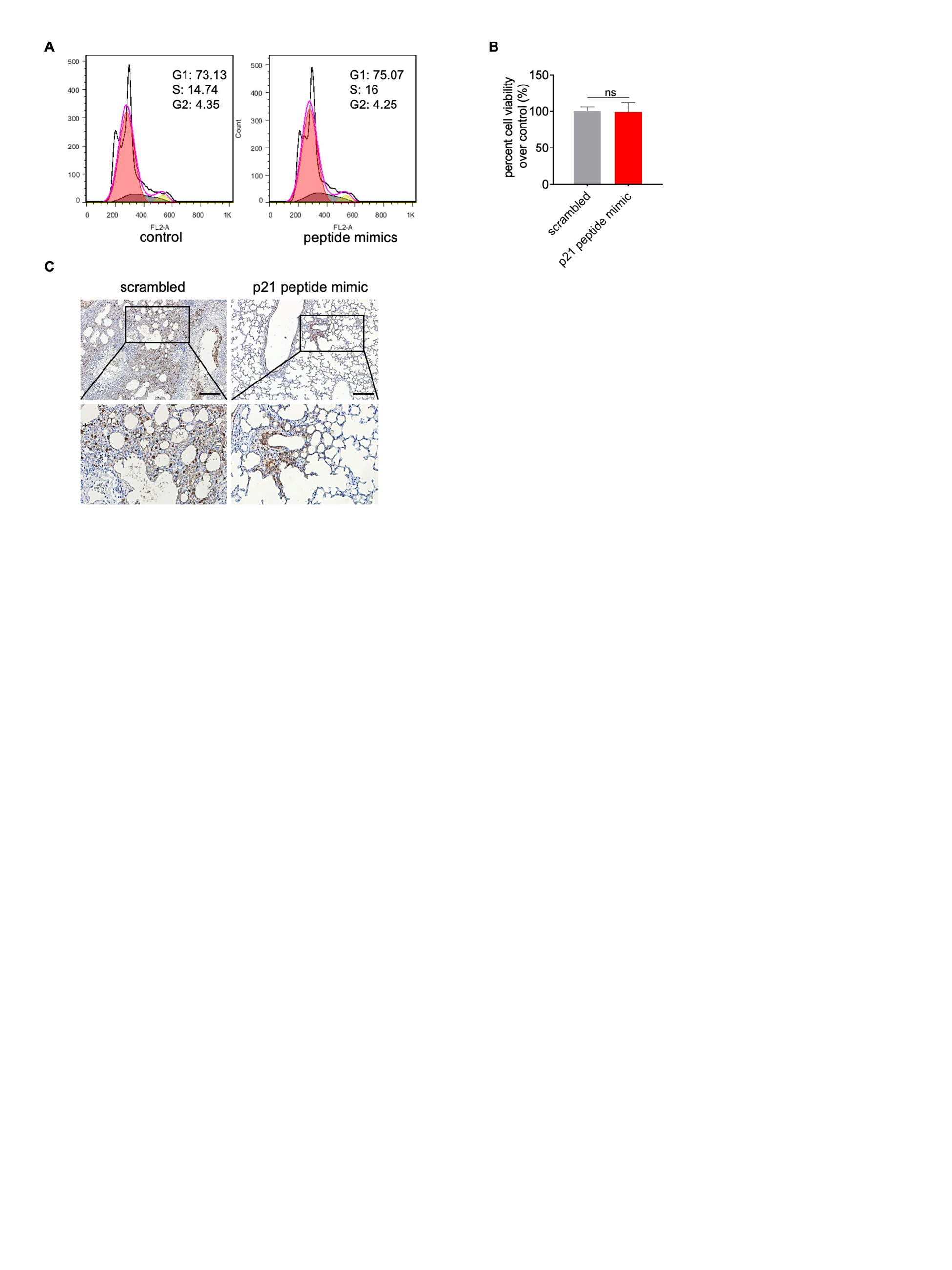

Supplement: S8 Fig — (A and B) A549 cells were treated with scrambled control peptides or peptide mimics. The cell cycle phase and growth rate were detected by flow cytometry and a CCK-8 kit, respectively. (C) C57/BL6J mice were intraperitoneally injected with p21 peptide mimics or scrambled peptides every other day and challenged with AH1 virus at 50 TCID50 (n = 6 mice in each group). Lung tissues were collected and analyzed by immunohistochemistry. Scale bar = 100 μm. All data are representative or presented as the mean ± SEM from three independent experiments unless specified. (TIF) [file ppat.1010295.s008.tif]
