# Supplementary material for: p21 restricts influenza A virus by perturbing the viral polymerase complex and upregulating type I interferon signaling
Source: PLoS Pathog. 2022 Feb 18;18(2):e1010295. doi: 10.1371/journal.ppat.1010295 (PMC8920271; doi:10.1371/journal.ppat.1010295)
Supplement: S1 Table — (DOCX) [file ppat.1010295.s010.docx]

**S1 Table. Primers used in this study.**

|  | Forward primer region (5’-3’) | Reverse primer region (5’-3’) |
| --- | --- | --- |
| CDKN1A | CCTTGGCCTGCCCAAGCTCTAC | GCGAGGCACAAGGGTACAAGA |
| NP | GGAATGGACCCCAGGATGTGCTC | TCTCCAGAAATTCCGGTCGTT |
| GAPDH | GTCTCCTCTGACTTCAACAGCG | ACCACCCTGTTGCTGTAGCCAA |
| IFN-β | ATGACCAACAAGTGTCTCCTCC | GCTCATGGAAAGAGCTGTAGTG |
| Mx1 | GGCTGTTTACCAGACTCCGACA | CACAAAGCCTGGCAGCTCTCTA |
| OAS1 | GGACTGAGGAAGACAACCAGGT | GGACTGAGGAAGACAACCAGGT |
| ISG15 | CTCTGAGCATCCTGGTGAGGAA | AAGGTCAGCCAGAACAGGTCGT |
